# Single-Cell Analysis with Spatiotemporal Control of Local pH

**DOI:** 10.1021/acsmeasuresciau.4c00079

**Published:** 2025-01-06

**Authors:** Kelsey Cremin, Gabriel N. Meloni, Orkun S. Soyer, Patrick R. Unwin

**Affiliations:** 1 Bio-Electrical Engineering Innovation Hub, University of Warwick, Coventry CV4 7AL, United Kingdom; 2 Department of Chemistry, University of Warwick, Coventry CV4 7AL, United Kingdom; 3 Molecular Analytical Science Centre for Doctoral Training, University of Warwick, Coventry CV4 7AL, United Kingdom; 4 School of Life Sciences, University of Warwick, Coventry CV4 7AL, United Kingdom; 5 Institute of Chemistry, Department of Chemistry, University of São Paulo, São Paulo, São Paulo 05508-000, Brazil

**Keywords:** scanning ion conductance microscopy (SICM), nanopipettes, confocal laser scanning microscopy (CLSM), finite element method modeling, local delivery, HeLa cells

## Abstract

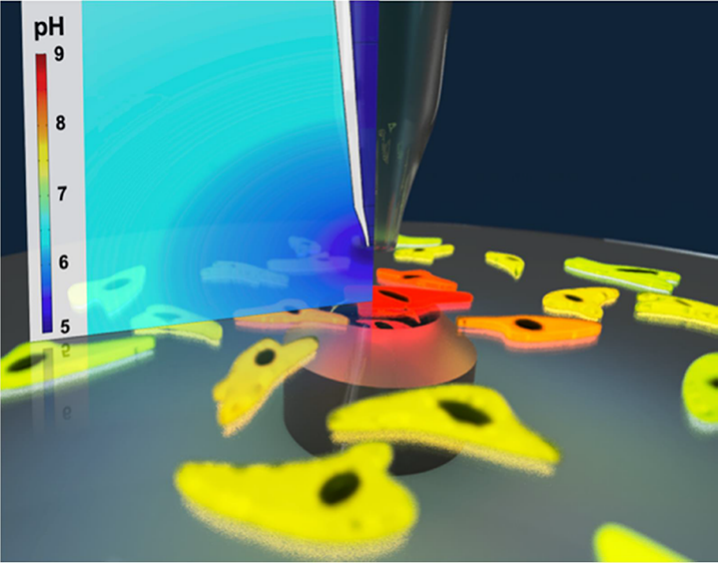

This work presents an experimental platform combining scanning ion conductance microscopy (SICM) with confocal laser scanning microscopy (CLSM), using intra- and extracellular pH indicator dyes to study the impact of acid delivery on individual HeLa cells within a population. The proton gradient generated by the SICM delivery is highly confined by the action of the media buffer, making the challenge local. Temporal and spatial aspects of the delivery are modeled by simulations, allowing for pH gradients across individual cells, even within a group, to be calculated. We find a strong dependency between the intracellular pH and the extracellular pH gradient imposed by local acid delivery. Postdelivery intracellular pH recovery depends on the extent of the acid challenge, with cells exposed to lower pH not returning to basal intracellular pH values after the extracellular pH recovers. This is a unique method for concentration-gradient challenge studies of cell populations that will have broad applications in cell biology. SICM can be used to deliver different chemicals and enables a wide range of local conditions to be applied across a cell population, for which the effects can be investigated at the single-cell level.

## Introduction

To comprehend cell heterogeneities and the role they may have within a cell community, single-cell measurement techniques are necessary.^1,2^ While many single-cell analysis techniques have been devised, the combination of direct single-cell measurement with control of the single-cell microenvironment (chemical composition and gradients) is achievable,^3,4^ but challenging. Design and use of tailored microfluidic devices, together with fluorescence or brightfield microscopy can sometimes allow combined control and measurement of the cell microenvironment. For example, bespoke devices have been devised for controlled drug-delivery and the generation of chemical gradients within such devices for combined analysis of cell behaviors, including chemotaxis.^5−8^ The microfluidics approach, however, is technically difficult, usually requiring a new microfluidic design for each analysis, and there are issues in loading and maintaining cells within devices.^9,10^ Thus, alternative methods for controlling microenvironments of single and small groups of cells would be beneficial.

One such method for localized delivery and analysis is scanning ion conductance microscopy (SICM).^11−13^ SICM uses a pipet as a scanning probe, and has primarily been developed for noninvasive physiochemical analyses of single cells, with subcellular resolution.^14−18^ SICM has been used for noninvasive studies of a range of interesting cell functions, including membrane permeability,^19−21^ membrane charge,^22−24^ and respiration.^25−27^ SICM and pipets have also been used to deliver compounds locally to both abiotic and biotic substrates.^28−30^ For charged species, the transport is controlled by migration under the electric field generated between the two quasi-reference counter electrodes (QRCEs), one in the pipet tip and one in the bathing solution, with additional contributions of diffusion and electroosmosis to consider. Therefore, a potential bias can be applied between the electrodes to promote or prevent the delivery of the charged species of interest (H^+^) from the tip lumen, and varying the potential allows for tunable control of the delivery flux.^30^ Simulating SICM delivery using finite element method (FEM) affords a predictive understanding of the spatial-temporal aspects of the SICM-imposed concentration gradient, allowing precise control of the chemical environment surrounding the pipet.^30−34^ The ability to generate well-defined concentration gradients across cells is of interest to study drug uptake,^35−37^ and a variety of other chemical interactions with cells.^38,39^ Thus, the key capabilities of SICM, namely the small scale, mobile tip, ease of use, amenability to modeling, and high control of delivery, makes it an ideal technique for creating controlled microscale environments around cells.

Here, we develop a single-cell analysis method by coupling SICM with confocal laser scanning microscopy (CLSM), and the use of appropriate intra- and extracellular fluorescent probes. This method draws on SICM delivery and FEM simulations to predictably change the chemical composition around individual cells, while the fluorescent probes report on cellular states. This approach allows for simultaneous evaluation of many individual cells across an image plane with the spatial distribution of the chemical challenge quantitatively described by FEM simulations, and with each cell exposed to a different concentration condition, affording high-throughput analysis of a range of local environments.

To demonstrate the combined SICM and fluorescence microscopy approach, we focus on the generation of external pH gradients around individual cells and cell groups using the HeLa cell line, a well-characterized model system used extensively in cancer research.^40^ The choice of a cancer cell line is particularly relevant as the pH dynamics are altered in many cancerous cell types, resulting in slightly higher intracellular pH, than for noncancerous cells, while the extracellular medium becomes more acidic, due to their different metabolism.^41,42^ We focused on disturbing this pH gradient across the cell membrane to demonstrate that the platform developed can be used to study pH regulation dynamics in individual cells.

## Results and Discussion

### A Combined SICM – CLSM System

**Figure 1:**
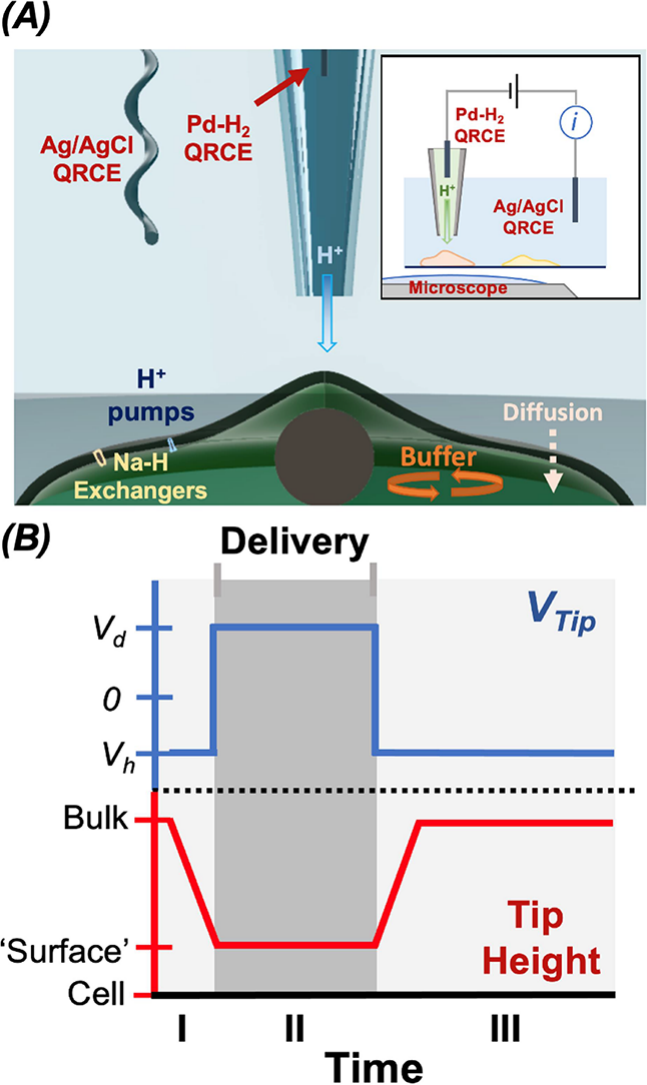
(A) Cartoon (not to scale) representing the combined CLSM and SICM approach, where the pipet tip is used to deliver acid in order to change the local pH near a cell. Some of the possible biological and physical processes that will impact the intracellular pH in response are shown. (B) Pipette potential (blue) and height (red) operated at each position in the delivery experiment. During time period I the pipet is approached toward the cell at a potential where protons are held inside the tip (V_h_). Once the approach feedback current threshold is reached, the potential is switched to the delivery potential (V_d_) for a set amount of time (time period II). After delivery, the potential is switched back to hold, V_h_, and the pipet is retracted to bulk (time period III). Further details are given in the Methods section.

Figure 1*A* illustrates the combined SICM and CLSM system. Cells are plated onto a dish with a coverslip glass base and observed with the inverted configuration CLSM, while the SICM probe height is controlled by an automated piezoelectric head, and the potential at the probe is controlled via custom electronics and a Field Programmable Gate Array (FPGA) card (see Methods). Local pH gradient generation is achieved by controlling the probe distance to cells together with the probe potential, which can be used to facilitate chemical delivery or uptake with high precision. A typical example pipet height/potential program is shown in Figure 1*B*, where the pipet is approached toward the surface of interest with an applied potential to retain the analyte (HCl) in the tip. Upon reaching the desired position, the potential is then changed to a value to release the analyte for a prescribed time after which the potential is reset, and the pipet is withdrawn from the surface before translation (in the *xy* plane) to the next position (cell) of interest. Experimental parameters for use in experiments were explored within FEM simulations (see Methods and SI). The effect of experimental conditions, including concentrations of delivered analyte, and probe pulse potentials, and their effect on gradient dynamics are explored in section SI-2.

### Generating and Visualizing Controlled Chemical (pH) Gradients

In our demonstrative example, we used the combined SICM-CLSM setup for generation of microscale pH gradients through delivery of protons (250 mM HCl in the pipet tip). Noncell specific pH-sensitive dyes fluorescein and 2′,7′-bis(2-carboxyethyl)-5-(and-6)-carboxyfluorescein (BCECF) were used to visualize the delivery from the pipet probe into culture media (M5 solution, see Methods). Fluorescein fluoresces green (510–520 nm) at neutral pH and fluorescence is reduced with acidification, as confirmed by the recorded fluorescence-pH calibration curve (see Supporting Information (SI), Figure S-7A).^43^ BCFL-AM is a cell permeant superior single-isomer formulation of BCECF, and was used for observing intracellular pH. BCFL-AM was calibrated with a spectrally analogous dye variant BCECF, which has the same p*K*_a_ as BCFL-AM but is not cell-specific,^44,45^ and can therefore be calibrated in solution. BCECF is a ratiometric dye that changes the ratio of emission at 535 nm for two excitation wavelengths (458 and 503 nm) depending on pH (calibration profile in Figure S-7B). For both dyes, fluorescence intensity was calibrated against pH in noninoculated culture media, and these calibrations were used to transform the experimental fluorescence intensity into pH values.

**Figure 2:**
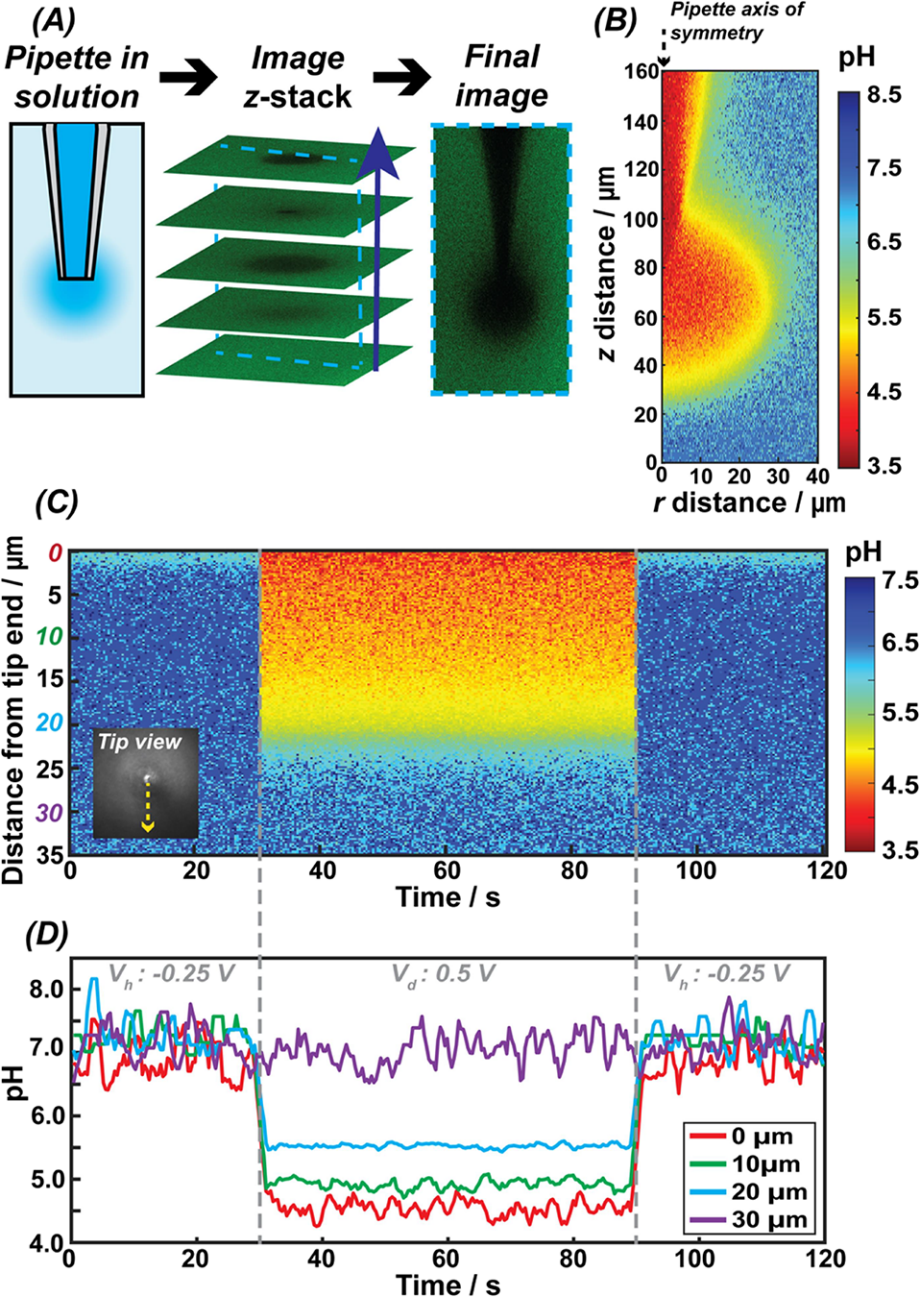
(A) Cartoon representation of the experimental setup, showing how z-stack imaging is performed traveling up the SICM probe during a delivery program. The z-stack is then reconstructed into a 3D projection, which can be positioned to create views as shown in the “Final image” panel. The combined SICM-CLSM application allows visualization of the pH gradient around the SICM probe in real time. (B) Steady-state heatmap showing pH around the probe during delivery of 250 mM HCl into M5 bath solution containing fluorescein (axisymmetric region shown), obtained by converting measured fluorescence into pH values. (C) Temporal heat map created by combining 400 z-stack images of a 9.70 μm wide strip normal to the end of the pipet lumen (shown as “tip view”), at 300 ms time intervals. (D) Line profiles of the fluorescein fluorescence intensity at different distances from the end of the pipet (from the heatmap in C). From 0 to 30 s: V_h_ = −0.25 V. From 30 to 90 s: V_d_ = 0.5 V. From 90 to 120 s: V_h_ = −0.25 V.

*Z*-stack imaging of the end of the pipet and the region external to it during acid delivery into an M5 solution containing fluorescein (*V*_*d*_ = 0.5 V) was used to visualize the delivery volume (Figure 2*A*). The presented image is a sideview projection of the 3D delivery volume, reconstructed from the z-stack images. Figure 2*B* shows the corresponding pH values, displaying a pH gradient extending from the pipet lumen out to the bulk solution, with the pH returning to bulk values (above pH 6.5) at an approximate distance of 40 μm from the lumen. This distance value agrees with FEM simulations of a probe delivering acid in bulk solution with the same experimental parameters (Figure S-5).

To visualize the pH change over time during delivery, a time lapse video was recorded for a smaller CLSM scan area (16 pixels wide strip of 9.70 μm) starting at the center of the micropipette lumen and extending outward, represented by the dashed line in Figure 2*C*. During the time lapse, the potential at the probe was varied to initiate delivery as follows: 30 s at the hold potential (*V*_*h*_ = −0.25 V); followed by 60 s at the delivery potential (*V*_*d*_ = 0.5 V); concluding with a 30 s at *V*_*h*_*.* The pH across the image at each measured time point is shown in Figure 2*C*. At the start of the delivery potential period (30 s), the pH rapidly decreases, and a stable pH gradient is established. When the hold potential is applied again at the end to switch off the pH challenge, the pH is almost instantly buffered back to bulk conditions. The rapid pH change across time is shown in Figure 2D at selected distances, which mirror the behavior shown in Figure S-6B simulation and highlight the ability to dynamically control the chemical environment around the pipet probe, via the applied tip potential.

Taken together, these results show that a combined SICM-CLSM approach allows highly accurate and temporally controllable generation, and analysis, of chemical gradients across micro scales.

### Analysis of Single Cell Behaviors in Local pH Gradients

To demonstrate the use of combined SICM-CLSM for cellular analysis, we generated local pH gradients around single HeLa cells while synchronously monitoring the intracellular pH of the cells. To measure external pH local to each cell, we used fluorescein-labeled wheat germ agglutinin (WGA), which binds to the cell surface and reports extracellular pH at the cell membrane.^46^ To measure intracellular pH we used pHRodo Red, which accumulates in the cytosol and has an increased fluorescence with decreasing pH. Using extra- and intracellular pH probes allows us to monitor the ability of a cell to maintain an internal pH in face of externally applied pH gradients. The experiments followed the framework set by the FEM simulation and pH-gradient measurements explained above.

**Figure 3:**
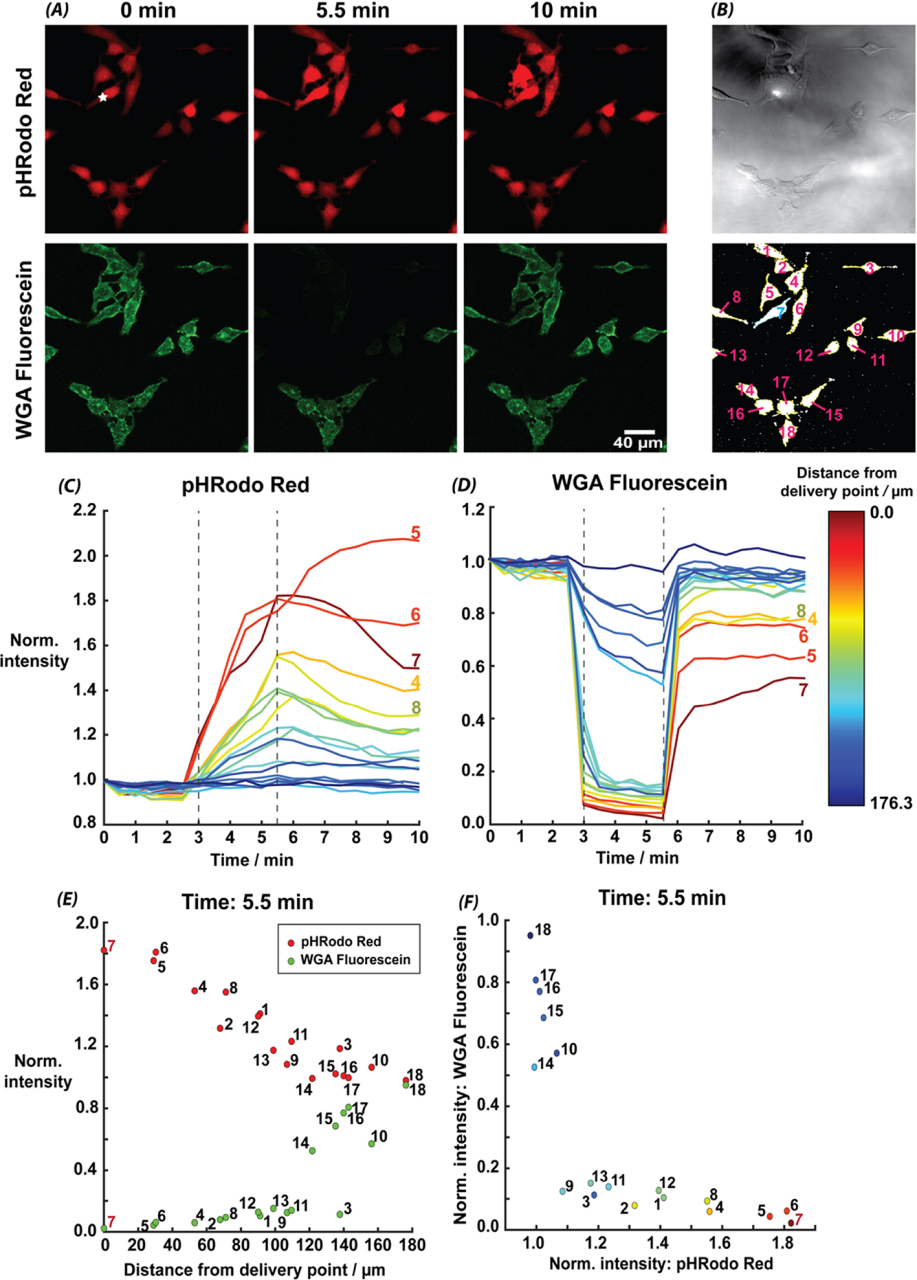
(A) Frames from CLSM time-lapse of HeLa cells at the beginning of the experiment (0 min), at the end of the delivery pulse (V_d_ = 0.5 V, time 5.5 min), and at the end of the experiment (10 min), from the pHRodo Red and WGA fluorescein channels. Total number of image frames = 20, with 30 s between frames. HeLa cells were held in an M5 media solution, with 250 mM HCl in the tip. (B) Brightfield image and ROI masks, showing the pipet halo directly above Cell 7. (C) pHRodo Red and (D) WGA fluorescein fluorescence intensity as a function of time, normalized to the intensity at 0 min, for each cell in the time lapse, where the two dashed lines represent the start and end of the delivery period (3–5.5 min). (E) Normalized intensity as a function of cell position from delivery point for both dyes at the end of the delivery pulse (5.5 min), and (F) the relationship between the response of the two dyes at the end of the delivery pulse (5.5 min), colored by cell position from the delivery point.

The SICM tip was approached and then held above a target cell at the hold potential (*V*_*h*_ = −0.25 V) for an initial period of 3 min, to measure the background fluorescence used to normalize the intensity across all other imaging frames in the time-lapse. The potential was then switched to delivery mode (*V*_*d*_ = 0.5 V) for 2.5 min, before returning to the hold potential during which cells were further imaged for 4.5 min (10 min of total imaging). Images from different time points are shown for the two dyes in Figure 3*A*, with the cell targeted by the probe marked with a white star. Figure 3*B* shows regions of interest (ROIs) masked for each cell identified in the images. These are used to label and track each individual cell across the time-lapse. The targeted cell (Cell 7) is seen below the halo of the tip in the brightfield image.

The normalized mean fluorescence intensity of pHRodo Red (Figure 3*C*), and WGA fluorescein (Figure 3*D*), for each cell is shown as a function of time, colored relative to the distance from the centroid of the target cell (Cell 7). The gray vertical dashed lines indicate the start and end of the acid delivery.

As seen in Figure 3*A* and 3*C*, the intracellular fluorescence intensity (pHRodo Red) increases in response to the acid delivery, and a significant response is seen in the cluster of cells surrounding the target cell (Cells 4 to 8), with the response becoming less pronounced further from the cell labeled 7 (delivery point). Beyond 140 μm from Cell 7 (blue shade in Figure 3*C*), the fluorescence does not significantly change across the entire experimental time frame, in agreement with the FEM-simulated reach of the acid gradient generated (Figure S-5).

During the recovery phase, following acid delivery, after the 5.5 min mark, cells 5 and 7, which are closest to the point of delivery, can be seen (Figure 3*A*) to show significant morphological changes, including some blebbing. This suggests that the most extreme pH challenge results in damage to the cells. The intracellular fluorescence for cell 5 continues to increase following the acid delivery, while for cells 6 and 7 the decrease in fluorescence values is much smaller, at the same period, than for other cells. We believe that cell damage at these extreme pH values means that the cell is unable to modulate the intracellular pH. The intracellular fluorescence for cells further from to the probe (cells 4, 6 and 8) begins to decrease more markedly over time (Figure 3*C*). These cells show recovery toward the initial fluorescence signal (intracellular pH) and no apparent morphological change across the time-lapse experiment.

The evident recovery in intracellular pH is most likely due to cellular mechanisms that exist to maintain controlled pH homeostasis, therefore acting against the pH challenge. Proton pumps, such as the vacuolar-type H^+^-ATPase, are upregulated and expressed in the plasma membrane of many cancer cells, and are used to remove acid from cells and maintain the pH balance.^47−49^ The activity of proton pumps and similar ion transporters are sensitive to changes in the pH environment, and will become more active in response to the pH challenge, regulating the intracellular pH.^50^ The response to the acidification may vary depending on the type of cancer cell and also as a result of epigenetic/genetic heterogeneity within a cancer type.^51,52^ By addressing the response of the individual cells, our proposed methodology is capable of highlighting these heterogeneous behaviors, for example, by comparing cells with similar local pH. This would require the accumulation of larger data sets than studied herein and could be a target for future studies.

Figure 3*D* shows that the extracellular pH at the targeted cell locality (WGA fluorescein channel) decreases during the delivery period, as expected, and returns to near prestimulus levels once delivery has stopped. The recovery of the WGA fluorescein fluorescence is much quicker than that of the intracellular pHRodo Red (panel C), due to efficient buffering and diffusion in solution, however there is still a distance dependence of the recovery, and cells subjected to the largest pH challenge have a longer recovery time and their fluorescence does not reach the initial levels across the experimental time length.

For the most aggressive region of challenge, the effect seen may result from nonrecoverable cell damage. Investigating the impacted processes and pathways within the cancer cells is beyond the scope of this work, but literature suggests how cells are influenced through acidification.^53,54^ Intracellular acidification can disrupt processes of cellular metabolism, such as glycolysis and the citric acid cycle, by altering the activity of enzymes.^53,55,56^ If extreme enough, the acid-induced altering of the cellular metabolism can result in ATP depletion and cell death. Acidification can also increase the production of harmful reactive oxygen species, or directly trigger apoptotic signaling pathways, initiating programmed cell death.^57,58^

The distance-dependence in pH response is highlighted in Figure 3*E*, which shows the normalized fluorescence intensity for both dyes at the end of the delivery period (5.5 min). Both dyes are responsive to the distance from the delivery point, confirming again that a pH gradient is established across the image plane. Figure 3*F* plots the normalized intensities of each dye (at 5.5 min) against each other, for each cell, with the color indicating distance from the delivery point. For cells beyond 140 μm (blue shades), fluorescein intensity shows a 5–20% change, while pHRodo Red only changes slightly (up to 5%). This confirms that at these distances the pH challenge is not significant enough to impact the intracellular pH by much across the delivery pulse length.

### Relating Intracellular pH Responses to Local pH Gradients

Quantitative data on the intracellular pH during delivery was acquired with BCFL-AM, a ratiometric intracellular pH dye, where the ratio between the emission at two distinct excitation wavelengths (I_458 nm_/ I_503 nm_) can be used to calibrate fluorescence to pH. A 1 μm radius pipet loaded with 250 mM HCl was used to deliver acid for 2 min to the target cells, with 2 min of imaging predelivery for establishing the background fluorescence. *Z*-stacks were used to image the majority of the cell volume, each comprising of three 2.5 μm interval slices (total volume of 7.5 μm). The time-lapses consisted of 40 images, separated by 15 s intervals (allowing 11.6 s of dark time between each exposure).

**Figure 4:**
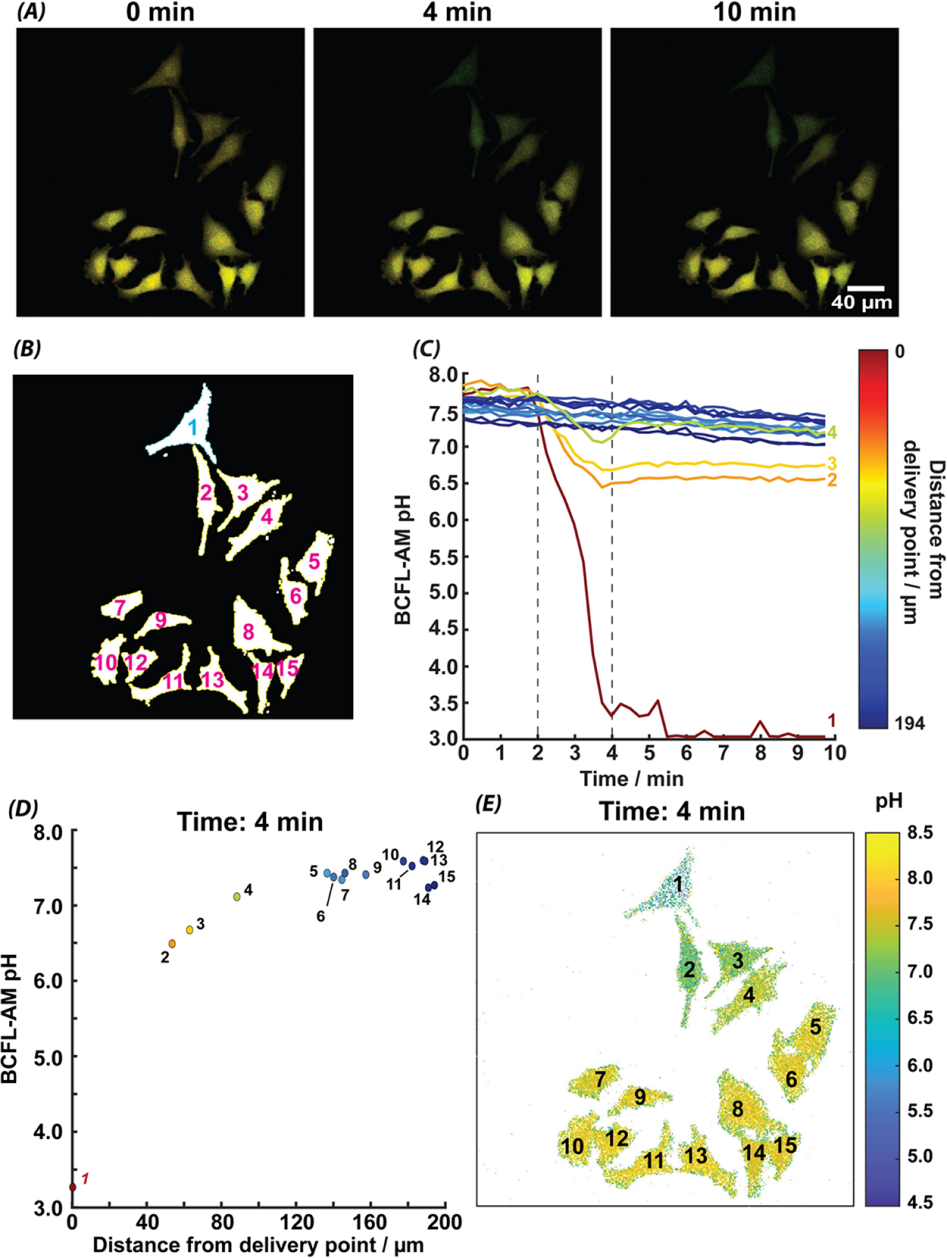
Delivery from a 1 μm radius pipet loaded with 250 mM HCl, to HeLa cells held in a bath solution of M5 media (A) Frames from CLSM time-lapse of HeLa cells at the beginning of the experiment (0 min), at the end of the delivery pulse (V_d_ = 0.5 V, 4 min), and at the end of the experiment (10 min), merged for the two excitation channels of BCFL-AM (green- 458 nm, red- 503 nm), and (B) labeled cells in the ROI masks used for image analysis, with the targeted cell highlighted (Cell 1). (C) The pH transformed by BCFL-AM calibration over time for each cell in the time-lapse, where the two dashed lines represent the start and end of the delivery (2–4 min), and (D) BCFL-AM pH as a function of distance of each cell centroid from the delivery point. (E) The intensity image of the cells in this experiment at 4 min transformed to pH (white used to remove nonfluorescing background).

Figure 4*A* shows images at different time points of the delivery experiment with BCFL-AM, at the beginning of the time-lapse (0 min), at the end of the delivery (4 min) and at the end of the experiment (10 min). In these images the intensity of the two channels is combined, and contrast enhanced for illustrative purposes. All analysis was performed with the raw data (Figure S-8). The identified ROIs used for tracking each cell are numbered in Figure 4*B*, with Cell 1 directly under the pipet lumen. The BCFL-AM calibration (Figure S-7B) was used to visualize the intracellular pH at the end of the delivery pulse (4 min, Figure 4*E*). The background is removed and shown in white. No cell blebbing is apparent (Figure 4*A*), possibly due to the shorter delivery time and less intense imaging protocol. The most distant cells (Cell 5 to 15), not affected by the acid delivery (Figure 4*D*), show a pH of approximately 7.3–7.4, in agreement with the intracellular pH expected for cancer cells.^38^ Cells closer to the delivery site show a lower pH value, with Cell 2, the closest neighbor to Cell 1, having an intracellular pH of 6.5 after the delivery. Cell 1, as expected, shows the lowest intracellular pH value postdelivery, however there is some fluctuation in values across the pixels within the cell. We reason that this can be caused by the sigmoidal response of the BCFL-AM ratiometric dye, which is very flat near these low pH values (see Figure S-7B).

Cell 1 showed the most dramatic change in pH during acid delivery and no recovery in pH beyond the delivery pulse. While close neighboring cells (Cell 2 to 4) were also strongly affected by the delivery (with a gradient effect with distance), their intracellular pH does return slightly in the first minutes postdelivery, stabilizing at pH values approximately 1 unit lower than the original level. This suggests a “point of no return” where the cell does not immediately recover, even without evident cell death (lack of morphological changes) and could be further investigated using other fluorescent probes such as those for signifying apoptosis. Even at the end of the delivery pulse, beyond *approx.* 140 μm from the delivery site, there is little effect observed on the intracellular pH (Figure 4*D*). This aligns with FEM simulations of this experimental setup (Figure S-5).

### High-Throughput Analysis of Single Cells Under Local pH Challenge

**Figure 5:**
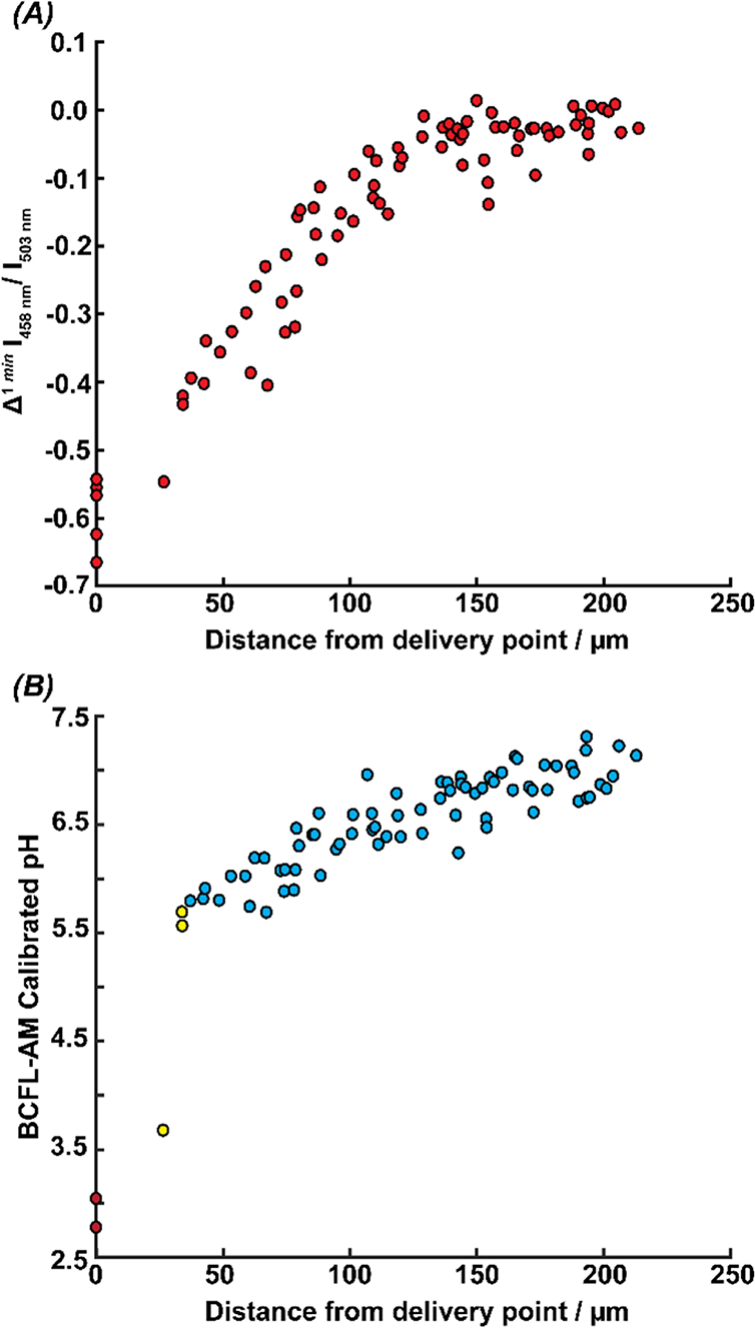
Clustered data from 5 individual delivery experiments performed in succession, where each point presents the response of one cell (87 cells in total). (A) Linear rate of change in BCFL-AM intensity across the first minute of delivery (Δ^1 min^ I _458 nm_/ I _503 nm_) as a function of cell position from the delivery point. (B) BCFL-AM pH calibration at the end of the delivery pulse (4 min) as a function of cell position from the delivery point. The marker color indicates the grouped pH environment of cells, based on COMSOL simulations. Red represents the cells exposed to a pH < 5, yellow to those in a pH environment between 5.5 and 6, and blue being those in an environment of pH > 6.5.

The tight control of pH gradient generation with SICM, together with the highly localized nature of generated gradients allows the acid delivery experiments to be repeated multiple times at different locations in the same culture dish. Exploiting this aspect of the methodology, we performed five individual time-lapse experiments in sequence at well-distanced points of the same sample across a 90 min window, in total capturing 87 different cells exposed to a pH challenge. Results from these experiments are collated in Figure 5. This framework allows the analysis of a sizable number of cells while each cell is considered as a single entity, allowing for heterogeneous behavior to be identified and further explored, something that is lost in averaging in population-based methods Each experiment was performed under the same delivery conditions, with a 2 min delivery of 250 mM HCl from the pipet tip, and the time-lapse was then continued up to 10 min postdelivery to record cell recovery or further changes. To help identify any trends in cell response to the acid delivery, we analyzed the linear rate of change of the normalized florescence ratio of BCFL-AM across the first minute of acid delivery for each cell (Δ^1*min*^ I_458 nm_/I_503 nm_), which could correlate to how dynamically cells respond to the challenge.

In Figure 5*A* larger negative values Δ^1 *min*^ I_458 nm_/ I_503 nm_ mean a faster decrease in BCFL-AM intensity values with the pH challenge. Cells closer to the delivery point have a more negative Δ^1 *min*^ I_458 nm_/ I_503 nm_, signifying a quicker acidification of the intracellular medium. Beyond 140 μm, the rate of change plateaus as the pH challenge is not sufficient to alter the cell intracellular pH, similar to what was seen in Figure 3*E*. Other metrics of interest regarding this experiment can be found in the SI (Figure S-9). This includes an examination of the cell response rate (normalized florescence ratio of BCFL-AM), in the first 2 min postdelivery, as a function of the distance of the cell from the delivery point.

The method described herein allows for high cell throughput analyses, at the single cell level. Considering that there may be heterogeneity in responses to the local pH challenge within a population of cells, it is important to point out that cell heterogeneity can be identified by this method to some extent (outlier responses) and then further explored. The cell parameters in the accompanying FEM simulations can also be varied, allowing us to further investigate the ranges of responses that might be due to certain heterogeneity (e.g., cell size, but also other parameters, with complementary single cell analyses).

### Tuning Cell-Local pH Gradients with SICM Parameters

We analyzed gradient dynamics induced by our SICM setup with further FEM simulations. These revealed that the pH gradient imposed by the probe is very steep, changing about 1–1.5 pH units within the first 20 μm from the delivery point and then terminating abruptly due to buffering effects of the media (Figure S-6B). As HeLa cells have an *approx*. dimension of 20 μm radius, this makes it difficult to “place” cells at very different local pH values. This is evident also in our experimental data, where intracellular pH responses are clustered (Figure 4*D*).

## Conclusions

We have introduced a platform to dynamically induce controlled concentration (pH) gradients across substrates with SICM while monitoring the response using fluorescent probes and CLSM. We have demonstrated the use of this framework for cellular studies, by delivering acid to cultured HeLa cells and monitoring changes in extra- and intracellular pH over time.

It should be noted that the focus of this work was on the development and demonstration of a measurement system, and so we did not employ a noncancerous biological control. Yet, the methodology does have in situ reference cells which are unperturbed during our experiments. These are the cells captured in the image plane, but at a considerable distance from the probe that they are not affected by the acid delivery. This aspect of the method was demonstrated by the consistent fluorescence response of these cells across the acid delivery experiments and agrees with the FEM model that cells at this distance are unperturbed. This aspect of the protocol is particularly useful for internal calibration and for identifying imaging artifacts, such as photobleaching.

FEM simulations of the SICM delivery and resulting spatial-temporal distribution for different experimental parameters allowed the fine-tuning of the delivery conditions and a predictive understanding of the pH challenge, which was corroborated with fluorescence intensity and the pH calibration of fluorescent probes. The rate and extension of intracellular pH change was found to depend on the distance of cells from the delivery site, and hence their position within the pH gradient.

The study of dynamics of pH homeostasis under local acid gradients can help to elucidate the active investment of a cell to maintain homeostasis against changing environments.^59^ Some treatments are being designed to disturb the pH imbalance found in cancer, using membrane transporters to disrupt the pH difference to induce apoptosis.^42,60,61^ We envisage systems where these treatments or other compounds of interest could be delivered, either to bulk or locally using a multibarreled probe system,^30^ and the cell is then exposed to a chemical gradient to measure the change in response across a population of cells. Beyond the studies of cancer, this platform could be applied to study other cell lines or biological sample types, such as tissue samples. The presented SICM-CLSM platform could readily be expanded to other chemical gradients, which would be informative for understanding cell-molecule interactions, cell–cell communications, or exploring metabolic pathways involved in response to different chemical gradients. The platform should be of interest as a means of investigating biotic and abiotic entities. The ability to visualize a large area while challenging and monitoring individual entities within a substrate provide a high throughput method, expanding the application of the method beyond biology.

## Methods

### Finite Element Method Simulation

COMSOL Multiphysics v. 5.6, with the Transport of Diluted Species and Electrostatics modules, was used for all simulations. Models consisted of a 2D axisymmetric cylindrical geometry of the experimental system representing the pipet positioned above an ellipsoidal cell. Two solution domains were simulated in the models, one inside the probe consisting of the acid solution, and the surrounding one representing the M5 media and accounting for the buffer speciation. A schematic of the model environment, along with additional simulation details can be found in the SI (SI-1)

### SICM Protocol

SICM pipets used for this work typically had a radius of 1 μm and were fabricated according to the procedure described in the SI (SI-6). A Ag/AgCl quasi reference counter electrode (QRCE) was used as the bulk electrode, as described previously,^62^ while the pipet QRCE was a palladium hydrogen (Pd–H_2_) electrode, as this maintained a more stable potential in highly acidic environments.^63^ The use of different electrodes and different concentration conditions in the pipet and bath, resulted in a potential difference between the two electrodes. This was always measured and accounted for by applying a potential offset of equivalent magnitude but opposite sign, such that no current flowed at the resulting potential.^64^ All potentials quoted herein are with respect to this initial condition.

The SICM protocol was performed as follows. Initially, the CLSM was used to position the probe in bulk solution (away from the cell surface) with the tip lumen positioned directly over the cell targeted for delivery. The image frame was selected to contain several cells at different distances from the pipet, where distant cells would remain unperturbed by the delivery and could be used as a control and for background correction. The pipet potential was set to result in appreciable (above noise) ionic current used as the SICM topography feedback, and to hold protons inside the micropipette (*V*_*h*_ > −0.2 V); as verified experimentally and through FEM simulations. The SICM pipet was then approached toward the cells (Figure 1*B*, Time I) until the current decreased by a determined threshold value (2%). A CLSM time lapse imaging sequence was then begun, and cells were imaged several times before delivery to record an initial cell fluorescence for comparison throughout the delivery experiment. The potential was then switched to deliver protons (*V*_*d*_ ≈ 0.5 V) to the targeted cell for a specified time (Figure 1*B*, Time II), following which the potential was returned to the approach potential (*V*_*h*_) and the probe was retracted into the bulk solution. The CLSM imaging continued for several minutes to follow the cell fluorescence postdelivery.

### Cell Culturing

HeLa cells (ECACC, catalogue number: 93021013) were grown and sampled as discussed in full in the SI (see SI-7). In short, cells were plated onto WillCo Wells coverslip glass dishes, and supplemented with Minimum Essential Medium (Sigma-Aldrich, 56416C, referred to here at M5) buffered with HEPES (100-fold dilution, Sigma-Aldrich, H0887). The M5 also served as the electrolyte for the SICM experiments and was characterized for the FEM simulation using MINEQL software (Table S-2).

### Confocal Laser Scanning Microscopy

Different fluorescent dyes were used to quantify the effects of acid delivery to cells and their surrounding environment, specifically fluorescein and 2′,7′-bis(2-carboxyethyl)-5-(and-6)-carboxyfluorescein (BCECF, Stratech Scientific Limited, 85138–49–4) to explore changes in the media pH, wheat germ agglutin (WGA) fluorescein (Invitrogen Molecular Probes, W834) which binds to glycoproteins and works as an indicator for the pH at the cell membrane/solution interface, and pHRodo Red (Invitrogen Molecular Probes, P35372) and BCFL-AM (AAT Bioquest, 21190) for recording the intracellular pH. The staining protocols used for these dyes and others explored in this study can be found in the Supporting Information (SI-8).

Cells and media fluorescence were measured across a z-stack time lapse series, with dark intervals between each scan of 15–30 s to allow the cells to rest and restrict photobleaching. Sparse *z*-stacks were used to capture most of the cell volume fluorescence. Details of the microscope and laser setting used in the confocal microscopy and explained in the SI (SI-8). Image analysis is described in SI-9.

## Data Availability

The authors confirm that the data supporting the findings of this study are available within the article and its Supporting Information, or upon further request from the corresponding authors.

## Supporting Information Available

FEM model, simulated pH gradients, pH-sensitive dye calibration, raw fluorescence images, further experimental analysis, pipet fabrication, HeLa cell culture and media preparation, confocal microscopy, image analysis (PDF)

## References

[ref1] Gay L.; Baker A. M.; Graham T. A. Tumour Cell Heterogeneity. F1000Research 2016, 5 (238), 238–15. 10.12688/f1000research.7210.1. PMC4776671 26973786

[ref2] Liang S. B.; Fu L. W. Application of single-cell technology in cancer research. Biotechnol. Adv. 2017, 35 (4), 443–449. 10.1016/j.biotechadv.2017.04.001. 28390874

[ref3] Chen K.; Yu R.; Li M.; Wang H.; Xie B.; Liu S.; Ying Y.; Long Y. In Situ Oxygen Generation via a Platinum-Based Wireless Nanopore Electrode for Single-Cell Manipulation. Small Methods 2024, e2401448 10.1002/smtd.202401448. 39428871

[ref4] Ying Y.-L.; Hu Y.-X.; Gao R.; Yu R.-J.; Gu Z.; Lee L. P.; Long Y.-T. Asymmetric Nanopore Electrode-Based Amplification for Electron Transfer Imaging in Live Cells. J. Am. Chem. Soc. 2018, 140 (16), 5385–5392. 10.1021/jacs.7b12106. 29529376

[ref5] Kim B. J.; Wu M. Microfluidics for mammalian cell chemotaxis. Ann. Biomed. Eng. 2012, 40 (6), 1316–1327. 10.1007/s10439-011-0489-9. 22189490 PMC3424276

[ref6] Wang B.; He B. S.; Ruan X. L.; Zhu J.; Hu R.; Wang J.; Li Y.; Yang Y. H.; Liu M. L. An integrated microfluidics platform with high-throughput single-cell cloning array and concentration gradient generator for efficient cancer drug effect screening. Mil. Med. Res. 2022, 9 (51), 1–17. 10.1186/s40779-022-00409-9. 36131323 PMC9494811

[ref7] Yahyazadeh Shourabi A.; Kashaninejad N.; Saidi M. S. An integrated microfluidic concentration gradient generator for mechanical stimulation and drug delivery. J. Sci.: Adv. Mater. Dev. 2021, 6 (2), 280–290. 10.1016/j.jsamd.2021.02.009.

[ref8] Hoppe T. J.; Moorjani S. G.; Shear J. B. Generating arbitrary chemical patterns for multipoint dosing of single cells. Anal. Chem. 2013, 85 (7), 3746–3751. 10.1021/ac4001089. 23427919 PMC3645469

[ref9] Chiu D. T.; deMello A. J.; Di Carlo D.; Doyle P. S.; Hansen C.; Maceiczyk R. M.; Wootton R. C. R. Small but Perfectly Formed? Successes, Challenges, and Opportunities for Microfluidics in the Chemical and Biological Sciences. Chem. 2017, 2 (2), 201–223. 10.1016/j.chempr.2017.01.009.

[ref10] Young E. W.; Beebe D. J. Fundamentals of microfluidic cell culture in controlled microenvironments. Chem. Soc. Rev. 2010, 39 (3), 1036–1048. 10.1039/b909900j. 20179823 PMC2967183

[ref11] Chen C. C.; Zhou Y.; Baker L. A. Scanning ion conductance microscopy. Annu. Rev. Anal. Chem. 2012, 5, 207–228. 10.1146/annurev-anchem-062011-143203. 22524219

[ref12] Hansma P. K.; Drake B.; Marti O.; Gould S. A. C.; Prater C. B. The Scanning Ion-Conductance Microscope. Science 1989, 243 (4891), 641–643. 10.1126/science.2464851. 2464851

[ref13] Page A.; Perry D.; Unwin P. R. Multifunctional scanning ion conductance microscopy. Proc. Math. Phys. Eng. Sci. 2017, 473 (2200), 20160889 10.1098/rspa.2016.0889. 28484332 PMC5415692

[ref14] Actis P.; Maalouf M. M.; Kim H. J.; Lohith A.; Vilozny B.; Seger R. A.; Pourmand N. Compartmental Genomics in Living Cells Revealed by Single-Cell Nanobiopsy. ACS Nano 2014, 8 (1), 546–553. 10.1021/nn405097u. 24279711 PMC3946819

[ref15] Actis P.; Tokar S.; Clausmeyer J.; Babakinejad B.; Mikhaleva S.; Cornut R.; Takahashi Y.; Lopez Cordoba A.; Novak P.; Shevchuck A. I.; et al. Electrochemical nanoprobes for single-cell analysis. ACS Nano 2014, 8 (1), 875–884. 10.1021/nn405612q. 24377306

[ref16] Happel P.; Thatenhorst D.; Dietzel I. D. Scanning ion conductance microscopy for studying biological samples. Sensors (Basel) 2012, 12 (11), 14983–15008. 10.3390/s121114983. 23202197 PMC3522950

[ref17] Novak P.; Li C.; Shevchuk A. I.; Stepanyan R.; Caldwell M.; Hughes S.; Smart T. G.; Gorelik J.; Ostanin V. P.; Lab M. J.; et al. Nanoscale live-cell imaging using hopping probe ion conductance microscopy. Nat. Methods 2009, 6 (4), 279–281. 10.1038/nmeth.1306. 19252505 PMC2702483

[ref18] Zhang J.; Zhu T.; Lang J.; Fu W.; Li F. Recent advances of scanning electrochemical microscopy and scanning ion conductance microscopy for single-cell analysis. Curr. Opin. Electrochem. 2020, 22, 178–185. 10.1016/j.coelec.2020.06.001.

[ref19] Chen C. C.; Zhou Y.; Morris C. A.; Hou J.; Baker L. A. Scanning ion conductance microscopy measurement of paracellular channel conductance in tight junctions. Anal. Chem. 2013, 85 (7), 3621–3628. 10.1021/ac303441n. 23421780 PMC3648657

[ref20] Huang K.; Zhou L.; Alanis K.; Hou J.; Baker L. A. Imaging effects of hyperosmolality on individual tricellular junctions. Chem. Sci. 2020, 11 (5), 1307–1315. 10.1039/C9SC05114G. 33209250 PMC7643560

[ref21] Zhou Y.; Chen C. C.; Weber A. E.; Zhou L.; Baker L. A.; Hou J. Potentiometric-scanning ion conductance microscopy for measurement at tight junctions. Tissue Barriers 2013, 1 (4), e25585 10.4161/tisb.25585. 24533255 PMC3805658

[ref22] Chen F.; He J.; Manandhar P.; Yang Y.; Liu P.; Gu N. Gauging surface charge distribution of live cell membrane by ionic current change using scanning ion conductance microscopy. Nanoscale 2021, 13 (47), 19973–19984. 10.1039/D1NR05230F. 34825684

[ref23] Klausen L. H.; Fuhs T.; Dong M. Mapping surface charge density of lipid bilayers by quantitative surface conductivity microscopy. Nat. Commun. 2016, 7 (12447), 1–10. 10.1038/ncomms12447. PMC5007656 27561322

[ref24] Perry D.; Paulose Nadappuram B.; Momotenko D.; Voyias P. D.; Page A.; Tripathi G.; Frenguelli B. G.; Unwin P. R. Surface charge visualization at viable living cells. J. Am. Chem. Soc. 2016, 138 (9), 3152–3160. 10.1021/jacs.5b13153. 26871001

[ref25] Ida H.; Takahashi Y.; Kumatani A.; Shiku H.; Matsue T. High Speed Scanning Ion Conductance Microscopy for Quantitative Analysis of Nanoscale Dynamics of Microvilli. Anal. Chem. 2017, 89 (11), 6015–6020. 10.1021/acs.analchem.7b00584. 28481079

[ref26] Simeonov S.; Schaffer T. E. High-speed scanning ion conductance microscopy for sub-second topography imaging of live cells. Nanoscale 2019, 11 (17), 8579–8587. 10.1039/C8NR10162K. 30994121

[ref27] Takahashi Y.; Zhou Y.; Miyamoto T.; Higashi H.; Nakamichi N.; Takeda Y.; Kato Y.; Korchev Y.; Fukuma T. High-Speed SICM for the Visualization of Nanoscale Dynamic Structural Changes in Hippocampal Neurons. Anal. Chem. 2020, 92 (2), 2159–2167. 10.1021/acs.analchem.9b04775. 31840491

[ref28] Babakinejad B.; Jonsson P.; Lopez Cordoba A.; Actis P.; Novak P.; Takahashi Y.; Shevchuk A.; Anand U.; Anand P.; Drews A.; et al. Local delivery of molecules from a nanopipette for quantitative receptor mapping on live cells. Anal. Chem. 2013, 85 (19), 9333–9342. 10.1021/ac4021769. 24004146

[ref29] Bruckbauer A.; James P.; Zhou D.; Yoon J. W.; Excell D.; Korchev Y.; Jones R.; Klenerman D. Nanopipette delivery of individual molecules to cellular compartments for single-molecule fluorescence tracking. Biophys. J. 2007, 93 (9), 3120–3131. 10.1529/biophysj.107.104737. 17631532 PMC2025666

[ref30] Page A.; Kang M.; Armitstead A.; Perry D.; Unwin P. R. Quantitative Visualization of Molecular Delivery and Uptake at Living Cells with Self-Referencing Scanning Ion Conductance Microscopy-Scanning Electrochemical Microscopy. Anal. Chem. 2017, 89 (5), 3021–3028. 10.1021/acs.analchem.6b04629. 28264566

[ref31] Chen B.; Perry D.; Page A.; Kang M.; Unwin P. R. Scanning Ion Conductance Microscopy: Quantitative Nanopipette Delivery-Substrate Electrode Collection Measurements and Mapping. Anal. Chem. 2019, 91 (3), 2516–2524. 10.1021/acs.analchem.8b05449. 30608117

[ref32] Cremin K.; Jones B. A.; Teahan J.; Meloni G. N.; Perry D.; Zerfass C.; Asally M.; Soyer O. S.; Unwin P. R. Scanning ion conductance microscopy reveals differences in the ionic environments of *Gram*-positive and negative bacteria. Anal. Chem. 2020, 92 (24), 16024–16032. 10.1021/acs.analchem.0c03653. 33241929

[ref33] Perry D.; Momotenko D.; Lazenby R. A.; Kang M.; Unwin P. R. Characterization of Nanopipettes. Anal. Chem. 2016, 88 (10), 5523–5530. 10.1021/acs.analchem.6b01095. 27108872

[ref34] Teahan J.; Perry D.; Chen B.; McPherson I. J.; Meloni G. N.; Unwin P. R. Scanning Ion Conductance Microscopy: Surface Charge Effects on Electroosmotic Flow Delivery from a Nanopipette. Anal. Chem. 2021, 93 (36), 12281–12288. 10.1021/acs.analchem.1c01868. 34460243

[ref35] Kilinc D.; Schwab J.; Rampini S.; Ikpekha O. W.; Thampi A.; Blasiak A.; Li P.; Schwamborn R.; Kolch W.; Matallanas D.; et al. A microfluidic dual gradient generator for conducting cell-based drug combination assays. Integr. Biol. (Camb.) 2016, 8 (1), 39–49. 10.1039/C5IB00209E. 26569638

[ref36] Shen S.; Zhang F.; Gao M.; Niu Y. Concentration Gradient Constructions Using Inertial Microfluidics for Studying Tumor Cell-Drug Interactions. Micromachines 2020, 11 (493), 493 10.3390/mi11050493. 32408585 PMC7281261

[ref37] Shi H.; Hou Z.; Zhao Y.; Nie K.; Dong B.; Chao L.; Shang S.; Long M.; Liu Z. Rapid and steady concentration gradient generation platform for an antimicrobial susceptibility test. Chem. Eng. J. 2019, 359 (2019), 1327–1338. 10.1016/j.cej.2018.11.046.

[ref38] Llopis J.; Mccaffery J. M.; Miyawaki A.; Farquhar M. G.; Tsien R. Y. Measurement of cytosolic, mitochondrial, and Golgi pH in single living cells with green fluorescent proteins. Cell Biol. 1998, 95, 6803–6808. 10.1073/pnas.95.12.6803. PMC22642 9618493

[ref39] Rismanian M.; Saidi M. S.; Kashaninejad N. A microfluidic concentration gradient generator for simultaneous delivery of two reagents on a millimeter-sized sample. J. Flow Chem. 2020, 10 (4), 615–625. 10.1007/s41981-020-00104-7.

[ref40] Mirabelli P.; Coppola L.; Salvatore M. Cancer Cell Lines Are Useful Model Systems for Medical Research. Cancers (Basel) 2019, 11 (8), 1098 10.3390/cancers11081098. 31374935 PMC6721418

[ref41] Cong D.; Zhu W.; Shi Y.; Pointer K. B.; Clark P. A.; Shen H.; Kuo J. S.; Hu S.; Sun D. Upregulation of NHE1 protein expression enables glioblastoma cells to escape TMZ-mediated toxicity via increased H(+) extrusion, cell migration and survival. Carcinogenesis 2014, 35 (9), 2014–2024. 10.1093/carcin/bgu089. 24717311 PMC4146414

[ref42] White K. A.; Grillo-Hill B. K.; Barber D. L. Cancer cell behaviors mediated by dysregulated pH dynamics at a glance. J. Cell. Sci. 2017, 130 (4), 663–669. 10.1242/jcs.195297. 28202602 PMC5339414

[ref43] Rudd N. C.; Cannan S.; Bitziou E.; Ciani I.; Whitworth A. L.; Unwin P. R. Fluorescence Confocal Laser Scanning Microscopy as a Probe of pH Gradients in Electrode Reactiosn and Surface Activity. Anal. Chem. 2005, 77 (19), 6205–6217. 10.1021/ac050800y. 16194080

[ref44] Marrone G.; De Chiara F.; Bottcher K.; Levi A.; Dhar D.; Longato L.; Mazza G.; Zhang Z.; Marrali M.; Fernandez-Iglesias A.; et al. The adenosine monophosphate-activated protein kinase-vacuolar adenosine triphosphatase-pH axis: A key regulator of the profibrogenic phenotype of human hepatic stellate cells. Hepatology 2018, 68 (3), 1140–1153. 10.1002/hep.30029. 29663481

[ref45] Intracellular pH Measurement with Dual Excitation Fluorescence Sensor BCFL; 2018. https://docs.aatbio.com/resources/assaywise/2018-7-2/2018-7-2.pdf.

[ref46] Zhao J.; Patwa T. H.; Lubman D. M.; Simeone D. M. Protein biomarkers in cancer: Natueral glycoprotein microarray approaches. Curr. Opin. Mol. Ther. 2008, 10 (6), 602–610. 19051138 PMC2920894

[ref47] Stransky L.; Cotter K.; Forgac M. The Function of V-ATPases in Cancer. Physiol. Rev. 2016, 96 (3), 1071–1091. 10.1152/physrev.00035.2015. 27335445 PMC4982037

[ref48] Cotter K.; Capecci J.; Sennoune S.; Huss M.; Maier M.; Martinez-Zaguilan R.; Forgac M. Activity of plasma membrane V-ATPases is critical for the invasion of MDA-MB231 breast cancer cells. J. Biol. Chem. 2015, 290 (6), 3680–3692. 10.1074/jbc.M114.611210. 25505184 PMC4319033

[ref49] McCarty M. F.; Whitaker J. Manipulating Tumor Acidification as a Cancer Treatment Strategy. Altern. Med. Rev. 2010, 15 (3), 264–272. 21155627

[ref50] Damaghi M.; Wojtkowiak J. W.; Gillies R. J. pH sensing and regulation in cancer. Front Physiol. 2013, 4 (370), 1–10. 10.3389/fphys.2013.00370. 24381558 PMC3865727

[ref51] Easwaran H.; Tsai H. C.; Baylin S. B. Cancer epigenetics: tumor heterogeneity, plasticity of stem-like states, and drug resistance. Mol. Cell 2014, 54 (5), 716–727. 10.1016/j.molcel.2014.05.015. 24905005 PMC4103691

[ref52] Yao J.; Czaplinska D.; Ialchina R.; Schnipper J.; Liu B.; Sandelin A.; Pedersen S. F. Cancer Cell Acid Adaptation Gene Expression Response Is Correlated to Tumor-Specific Tissue Expression Profiles and Patient Survival. Cancers (Basel) 2020, 12 (8), 2183 10.3390/cancers12082183. 32764426 PMC7463722

[ref53] Persi E.; Duran-Frigola M.; Damaghi M.; Roush W. R.; Aloy P.; Cleveland J. L.; Gillies R. J.; Ruppin E. Systems analysis of intracellular pH vulnerabilities for cancer therapy. Nat. Commun. 2018, 9 (2997), 1–11. 10.1038/s41467-018-05261-x. 30065243 PMC6068141

[ref54] Parks S. K.; Chiche J.; Pouyssegur J. Disrupting proton dynamics and energy metabolism for cancer therapy. Nat. Rev. Cancer 2013, 13 (9), 611–623. 10.1038/nrc3579. 23969692

[ref55] Lee Z. W.; Teo X. Y.; Song Z. J.; Nin D. S.; Novera W.; Choo B. A.; Dymock B. W.; Moore P. K.; Huang R. Y. J.; Deng L. W. Intracellular Hyper-Acidification Potentiated by Hydrogen Sulfide Mediates Invasive and Therapy Resistant Cancer Cell Death. Front Pharmacol. 2017, 8 (763), 1–10. 10.3389/fphar.2017.00763. 29163155 PMC5671507

[ref56] Boedtkjer E.; Pedersen S. F. The Acidic Tumor Microenvironment as a Driver of Cancer. Annu. Rev. Physiol. 2020, 82, 103–126. 10.1146/annurev-physiol-021119-034627. 31730395

[ref57] De Milito A.; Iessi E.; Logozzi M.; Lozupone F.; Spada M.; Marino M. L.; Federici C.; Perdicchio M.; Matarrese P.; Lugini L.; et al. Proton pump inhibitors induce apoptosis of human B-cell tumors through a caspase-independent mechanism involving reactive oxygen species. Cancer Res. 2007, 67 (11), 5408–5417. 10.1158/0008-5472.CAN-06-4095. 17545622

[ref58] Di Sario A.; Bendia E.; Omenetti A.; De Minicis S.; Marzioni M.; Kleemann H. W.; Candelaresi C.; Saccomanno S.; Alpini G.; Benedetti A. Selective inhibition of ion transport mechanisms regulating intracellular pH reduces proliferation and induces apoptosis in cholangiocarcinoma cells. Dig. Liver Dis. 2007, 39 (1), 60–69. 10.1016/j.dld.2006.07.013. 16982221

[ref59] Johnson C. G. M.; Fletcher A. G.; Soyer O. S. ChemChaste: Simulating spatially inhomogeneous biochemical reaction-diffusion systems for modeling cell-environment feedbacks. Gigascience 2022, 11, 1–12. 10.1093/gigascience/giac051. PMC9205757 35715874

[ref60] Almasi S.; El Hiani Y. Exploring the Therapeutic Potential of Membrane Transport Proteins: Focus on Cancer and Chemoresistance. Cancers 2020, 12 (6), 1624–1631. 10.3390/cancers12061624. 32575381 PMC7353007

[ref61] Hao G.; Xu Z. P.; Li L. Manipulating extracellular tumour pH: an effective target for cancer therapy. RSC Adv. 2018, 8 (39), 22182–22192. 10.1039/C8RA02095G. 35541713 PMC9081285

[ref62] Bentley C. L.; Perry D.; Unwin P. R. Stability and Placement of Ag/AgCl Quasi-Reference Counter Electrodes in Confined Electrochemical Cells. Anal. Chem. 2018, 90 (12), 7700–7707. 10.1021/acs.analchem.8b01588. 29808685

[ref63] Bentley C. L.; Kang M.; Unwin P. R. Nanoscale Structure Dynamics within Electrocatalytic Materials. J. Am. Chem. Soc. 2017, 139 (46), 16813–16821. 10.1021/jacs.7b09355. 29058886

[ref64] Perry D.; Page A.; Chen B.; Frenguelli B. G.; Unwin P. R. Differential-Concentration Scanning Ion Conductance Microscopy. Anal. Chem. 2017, 89 (22), 12458–12465. 10.1021/acs.analchem.7b03543. 28992688

