# Supplementary material for: Single-Cell Analysis with Spatiotemporal Control of Local pH
Source: ACS Meas Sci Au. 2025 Jan 6;5(1):120–9. doi: 10.1021/acsmeasuresciau.4c00079 (PMC11843512; doi:10.1021/acsmeasuresciau.4c00079)
Supplement: Supplementary file 1 — tg4c00079_si_001.pdf [file tg4c00079_si_001.pdf]

Supporting Information

Single cell analysis with spatiotemporal control of local pH

Kelsey Cremin[1-4], Gabriel N. Meloni [1,2,5], Orkun S. Soyer [1,4], Patrick R. Unwin[1,2*]*

[1] Bio-Electrical Engineering Innovation Hub, University of Warwick, Coventry CV4 7AL, United Kingdom.
[2] Department of Chemistry, University of Warwick, Coventry CV4 7AL, United Kingdom.
[3] Molecular Analytical Science Centre for Doctoral Training, University of Warwick, Coventry CV4 7AL, United Kingdom
[4] School of Life Sciences, at the University of Warwick, Coventry CV4 7AL, United Kingdom
[5] Institute of Chemistry, Department of Chemistry, University of São Paulo, São Paulo, SP 05508-000, Brazil

*Corresponding author(s):

p.r.unwin@warwick.ac.uk

o.soyer@warwick.ac.uk

Contents

SI-1 FEM model S.3

S-2 Simulated pH gradients S.7

S-3 pH sensitive dyes calibration S.11

S-4 Raw florescence images for Figure 4A S.12

SI-5 Further experimental analysis S.13

SI-6 Micropipette fabrication S.15

SI-7 HeLa cell culture and media preparation S.15

SI-8 Confocal microscopy S.15

SI-9 Image analysis S.17

SI-1 FEM model

All simulations were performed with COMSOL Multiphysics v. 5.6, using the Transport of Diluted Species and Electrostatics modules. Figure S-1 illustrates the 2D axisymmetric model with the pipette geometry taken from images of representative pipettes (Figure S-2A). The model includes an ellipsoid cell geometry on an inert surface (glass slide) under the pipette, approximating to the realistic volume of HeLa cells. A no flux condition was applied to the cell wall which is reasonable as this represents a very small source/sink compared to the pipette and solution. The pipette/cell separation (*d*) was changed to the experimental approach distance to simulate the delivery to cells (vide infra) or 100 μm to simulate the delivery to bulk solution. Detailed descriptions of the simulation domain and boundary conditions are given in Table S-1.

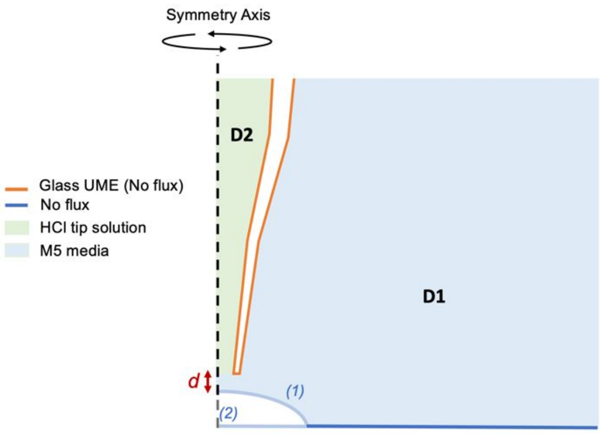

Figure S-1. *Schematic of the COMSOL model, with bulk solution containing M5 media (D1) and the pipette containing HCl for delivery (D2), at the approach distance (d). The geometry of the surface includes the outline of a HeLa cell.*

Transport of chemical species was reasonably assumed to be by diffusion and migration in the electric field generated between the two QRCEs. Although electroosmotic flow can contribute to mass transport at pipettes, even at high applied potentials,[1] the environment inside of the pipette is very acidic making the pipette wall surface charge density much smaller than in studies at physiological pH. Also, the high ionic strength of the acid solution and the relatively large pipette dimension, compared to nanopipettes typically used in SICM, significantly reduces the contribution of electroosmosis to the overall species flux[2-4] (see main manuscript discussion and Section S-2).

The flux J_i of each species (i), was described by the Nernst-Planck equation:

$$J_i = -D_i \nabla c_i - z_i \frac{F}{RT} D_i c_i \nabla \phi \quad \text{(S1)}$$

with the electric potential, ϕ, defined by the Poisson equation:

$$\nabla^2 \phi = -\frac{F}{\varepsilon \varepsilon_0} \sum_i z_i c_i \quad \text{(S2)}$$

where D_i, z_i and c_i are the diffusion coefficient, charge number, and concentration of a given chemical species i, respectively. F, R and T are the Faraday constant, gas constant and absolute temperature (298 K), ε is the relative permittivity of the solution (78), and ε_0 the vacuum permittivity (8.85×10^{-12} F m^{-1}). The diffusion coefficients (D_i) of all species are summarized in Table S-1. Diffusion coefficients are values at infinite dilution, and concentrations were not activity corrected. This was considered acceptable as the purpose of the model was to explore generally the effects of changing experimental parameters, and not to quantify pH changes. Rather, the calibrated fluorescent pH dyes were used to quantify experimental pH.

Table S-1. *Diffusion coefficient and concentration of all species included in COMSOL model*

Species	Diffusion coefficient (cm^2 s^{-1}) (CRC)	Initial Conc. in D1 (mM)	Initial Conc. in D2 (mM)
Na^+	1.33×10^{-5}	126.40	126.40
K^+	1.96×10^{-5}	4.98	4.98
Cl^-	2.03×10^{-5}	125	125
Ca^{2+}	7.92×10^{-6}	1.81	1.81
$HHEPES^+$	5.00×10^{-5}	$\frac{[HEPES] \times [H^+]}{K_{a_{HHEPES+}}}$	0
HEPES	5.00×10^{-5}	$\frac{[HEPES^-] \times [H^+]}{K_{a_{HEPES}}}$	0
$HEPES^-$	5.00×10^{-5}	10 to 10×10^{-3}	0
H^+	9.80×10^{-9}	0.0001	20 to 250
OH^-	5.27×10^{-5}	0.0001	$10^{-(pKw-pH)}$

M5 media used in these experiments is rather complex, containing many different species. MINEQL software was used for speciation of the media, and the major contributing

ions (>98% of total ions) where accumulated and balanced for inclusion in the model, while ignoring minor constituents for computational efficiency. The media was buffered with 10 mM HEPES buffer, which was also accounted for in the model. Table S-1 summarizes all species accounted for in the simulations.

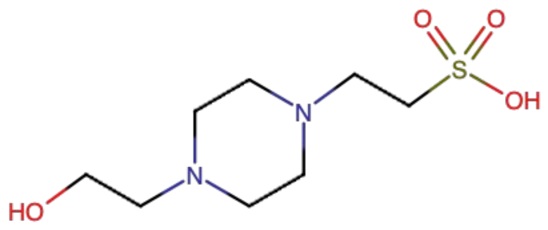

HEPES = $C_8H_{18}N_2O_4S$

The component reactions for the HEPES buffer are as follows:[5]

$$\mathrm{H}\mathbf{HEPES}^{+} \rightleftharpoons \mathrm{H}^{+} + \mathbf{HEPES} \qquad \mathrm{pK} \approx 3.0$$

$$\mathbf{HEPES} = \mathrm{H}^{+} + \mathbf{HEPES}^{-} \qquad \mathrm{pK} = 7.564$$

Figure S-2. *The chemical structure and component reaction for HEPES, the buffering component of M5 media used in SICM experiments.*

Buffer equilibria reactions were simulated as fast processes in domain D1 to ensure equilibrium was maintained.

The pipette-cell separation (*d*) is a key parameter to understand the pH distribution during tacid delivery to cells. The experimental separation is determined by simulating an SICM approach curve (Figure S-3B) of the pipette ionic current at the approach potential (V_h) for varying values of *d*. A 2% decrease of current compared to bulk was the experimental current threshold used as feedback to set the distance in all experiments.

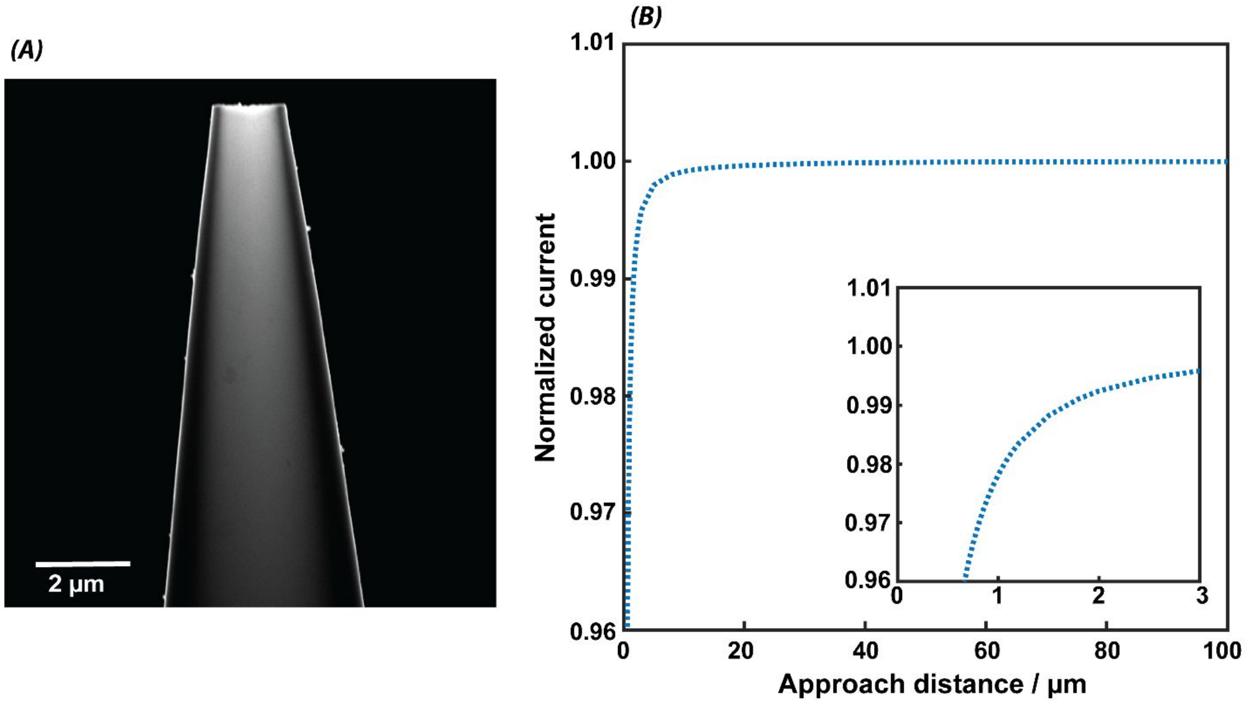

Figure S-3. *(A) STEM image of a typical pipette of the size used in the SICM experiments (lumen radius (r) = 0.8 µm), where geometries are used in the COMSOL model. (B) COMSOL simulated approach curve of pipette approaching the cell surface,* V_h *= -0.25 V, with the insert highlighting the region where the normalized current crosses the threshold value of 0.98 (1.05 µm).*

S-2 Simulated pH gradients

The pH field generated by acid delivery from a pipette *(r* = 1 μm) containing 250 mM HCl biased at V = 0.3 V into bulk solution is shown in Figure S-4. Two distinct steady-state simulations were run for the same delivery parameters, one disregarding the buffer reactions in D1 (unbuffered) and the other considering the reactions (buffered). The action of the buffering capacity on the delivered pH gradient is evident, constraining the pH field to a region near the pipette. Note that the unbuffered case results should not be taken quantitatively, especially near the boundary where the bulk pH was set as the condition. The external boundary in the unbuffered case is too close for this simulation, but the results serve to show to strong effect of buffer. The boundary was set to give a faithful representation for the buffered case.

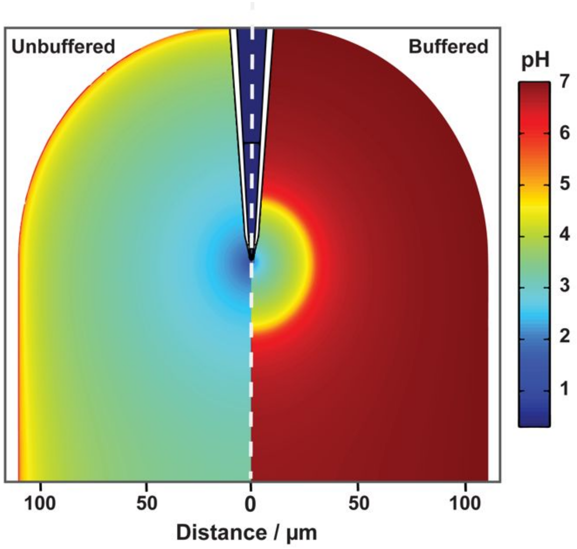

Figure S-4. *Simulated pH distribution for the delivery of 250 mM HCl (V_d = 0.3 V) from a pipette (r = 1 μm) into a bath of M5, containing 10 mM HEPES buffer, with either the buffer reactions disabled (left) and on (right). d = 100 μm.*

As described, the delivery profile and subsequent change in the pH can be altered by changing experimental conditions. Figure S-5 shows the effect of changing several experimental variables on the radial pH profiles from the pipette center in the *z*-direction: the HEPES buffer concentration (Figure S-5A); the HCl concentration in the pipette (Figure S-5B); and the pipette delivery potential (V_d) (Figure S-5C). Each condition was changed, with other parameters set to those used experimentally (buffer concentration = 10 mM, pipette HCl concentration = 250 mM, V_d = 0.5 V). All the purple traces in the three sub-plots are simulated with the same experimental conditions and are therefore identical.

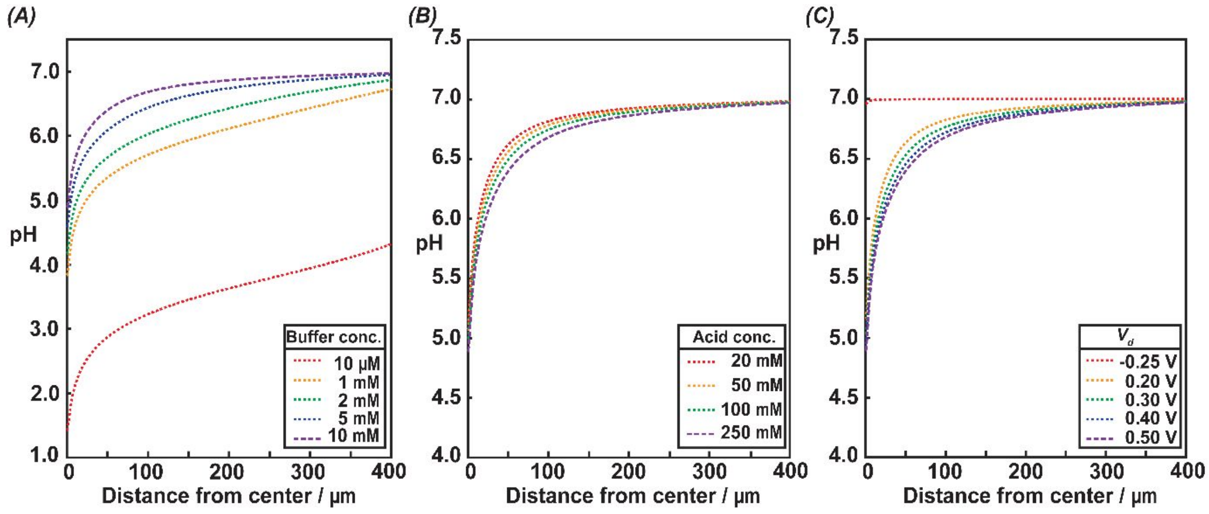

Figure S-5. *Simulated pH distribution from the micropipette center, moving in the z-direction away from the pipette opening, for different experimental conditions: (A) the pipette delivery potential (V_d), (B) the concentration of HCl in the pipette, and (C) HEPES buffer concentration. All results from steady-state simulations, with other parameters defined in the text.*

Figure S-5A shows the pH profiles generated by a pipette delivering when the HEPES buffer concentration is decreased from the standard concentration in the M5 media of 10 mM. Lower buffer concentrations result in pH gradients that extend further into solution, due to the diminished buffer capacity of the media. Figure S-5B shows the effect of changing the acid concentration in the pipette on the pH profile. For the acid concentration range considered, the effect of changing the HCl concentration (a strong acid) is relatively small compared to the effect of changing the buffer concentration (panel A). The effect of the delivery potential (V_d) on proton delivery is clearly demonstrated in Figure S-5C. From these simulations the experimental hold (V_h = *-0.25 V*) and delivery (V_d= *0.5 V*) potentials are established for the acid (250 mM) and buffer (10 mM) concentrations used. As expected, large positive potentials favour cation migration from the pipette, while negative potentials favour migration into the pipette.[6] There is little change in the pH profile beyond 0.4 V, and so 0.5 V was selected as the delivery potential. At -0.25 V protons are retained in the pipette, with the pH at a distance from the pipette equivalent to the experimental pipette/cell separation (1.05 µm, see Figure S-3) changing by only 1.48% from bulk values, and so this was selected as the hold (approach) potential.

Figure S-6A shows the steady-state simulated pH gradient generated by a 1 μm radius pipette, loaded with 250 mM HCl held at the approach height over a cell (d = 1.05 μm), under the selected delivery potential (V_d = 0.5 V). The inset is zoomed to the cell/pipette gap, highlighting the pH gradient experienced by the cell. Figure S-6B shows the time-dependent radial distribution of pH during (and after) a 120 s delivery pulse. The different distance values represent different distances from the pipette center (0 μm), taken at the approach distance (d = 1.05 μm - dashed line in Figure S-6A). The delivery pulse starts at 0 s and lasts for 120 s (V_d = 0.5V, blue background), after which the potential is returned to the hold potential (V_h = -0.25 V, white background) and held there for a further 120 s. The pH values reach a steady-value fairly quickly, within the delivery pulse, for all distances. Transport time and reaction with the solution buffer, accounts for the longer times to reach a stable value observed at larger radial distances. Nonetheless, a steady distribution is achieved within 15 seconds for all distances. The pH returns to bulk values within 20 s of the end of the delivery pulse as a result of the media buffer capacity. It is this quick establishment of a steady pH gradient that allows constant, quantifiable and easily changeable concentration gradients to be established with SICM, offering a major advantage over other techniques, such as microfluidics. The steady gradient eases the analysis of experimental results, as the concentration gradient can be considered constant whilst the delivery potential is held.

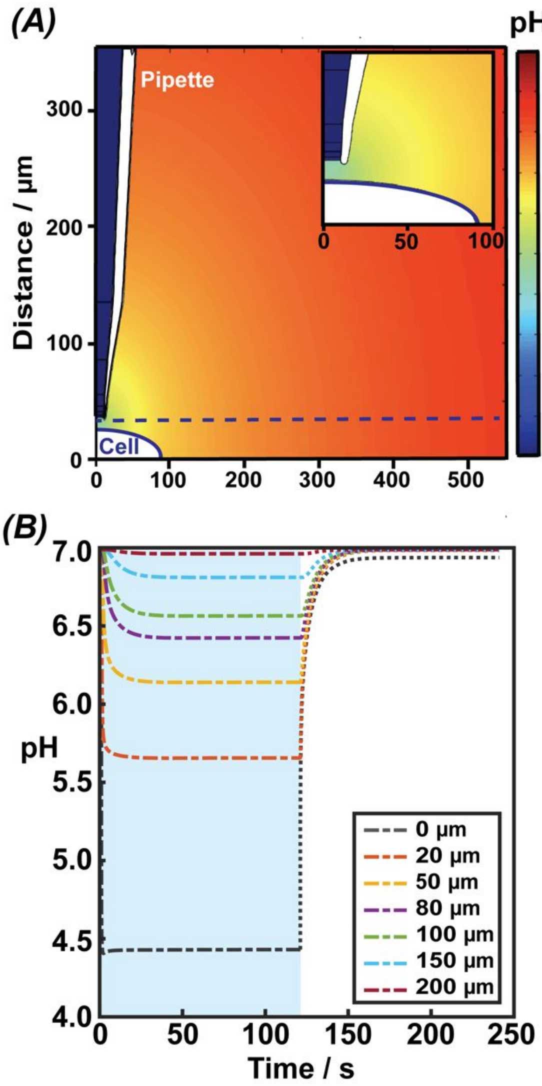

Figure S-6. *(A) Simulation domain, showing a steady-state study of the pipette delivering acid (250 mM, V_d = 0.5 V, d = 1.05 µm). Insert shows a zoom of the pipette/cell gap. (B) Time dependent simulation of a 120 s pulse delivering 250 mM HCl into buffered M5 media (V_d = 0.5V, blue background), followed by the returned to approach potential conditions (V_h = -0.25V, white background), at different distances from the pipette center following the dashed line in (A). Tip-cell distance = 1.05 µm, pipette radius = 1 µm.*

S-3 pH sensitive dyes calibration

A repeated calibration was performed by measuring the fluorescence intensity of M5 media solutions containing fluorescein, buffered to a series of pH values. The calibration plots are shown in Figure S-7A, and used to convert the fluorescein fluorescence intensity from Figure 2A of the main manuscript to pH values in Figure 2B and C.

BCECF (10 μM, Invitrogen, Catalog number: B1151), a non-cell specific analog to BCFL-AM, was calibrated in M5 media, buffered to different pH values. The resulting calibration series is shown in Figure S-7B, which was performed in triplicate for every pH value. The calibration curve is formed by applying a smoothed spline fit to the data. This calibration was used to convert the fluorescence measurements of BCFL-AM, in the cell experiments (Figure 4 and 5, main manuscript).

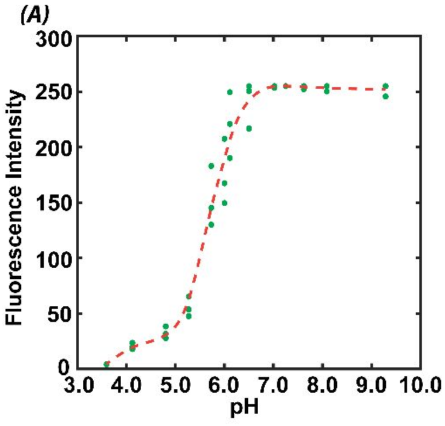

Figure S-7. *(A) pH calibration of fluorescein intensity (2 μM, Gain: 700V) and (B) pH calibration of BCECF normalized intensity ratio (10 μM, gain 800V), which was extended to experiments with BCFL-AM.*

S-4 Raw fluorescence images for Figure 4*A*

Figure S-8 complements Figure 4 in the main text, where the raw fluorescence intensities for each channel are shown. The emission for the excitation at 458 nm is shown in green, and the emission for the excitation at 503 nm is shown in orange.

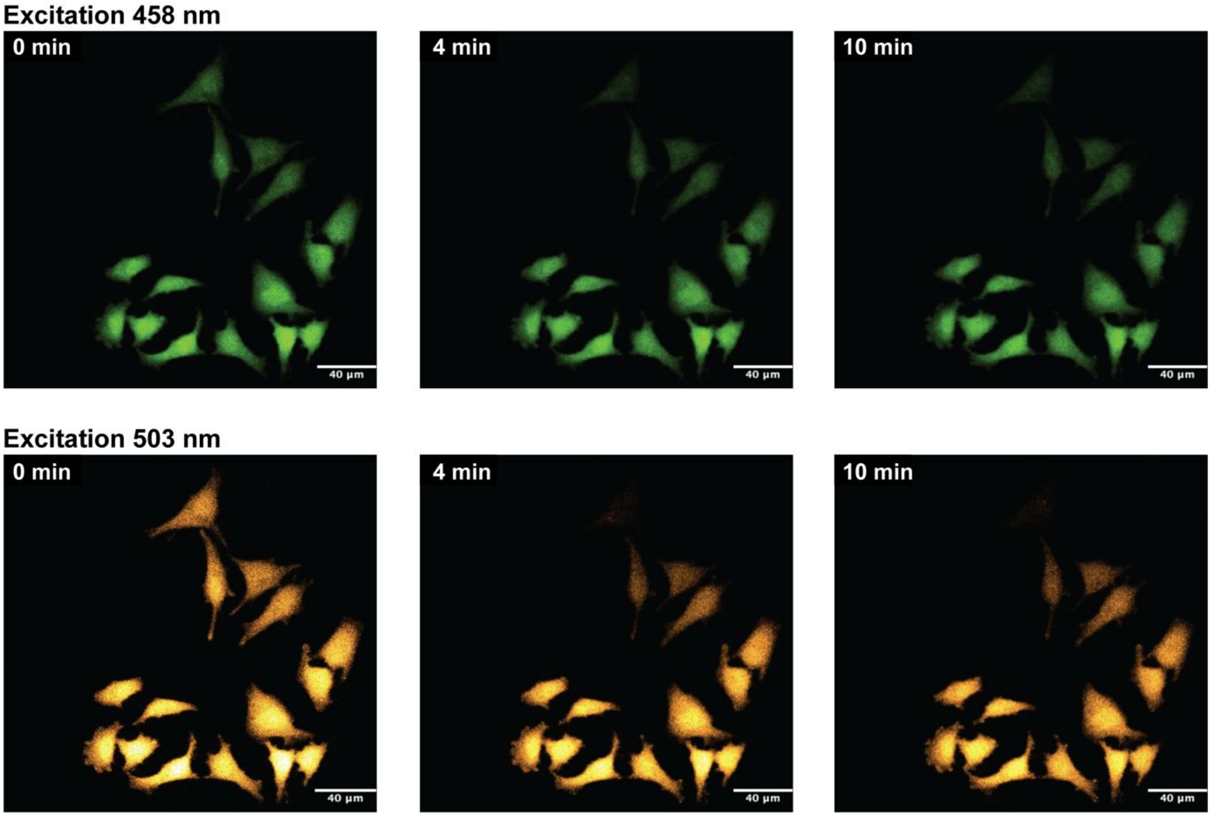

Figure S-8. *Fluorescence images for delivery experiment to HeLa cells at the beginning of the time lapse (0 min), at the end of the delivery pulse (V_d = 0.5 V, for 2 min) (4 min), and at the end of the time lapse (10 min), for the two excitation channels of the ratiometric BCFL-AM dye (green- 458 nm, orange- 503 nm).*

SI-5 Further experimental analysis

Figure S-9A shows the mean $I_{458\ nm}/ I_{503\ nm}$ for BCFL-AM across each cell at the end of the delivery pulse (4 min) against distance from the delivery site for every cell in the experiment portrayed in Figure 5. These intensity ratio values were converted to pH using the calibration in Figure S-7D. Similar to the data in Figure 3 and 4, there is a clear trend of the degree of cell acidification and distance from the delivery position, with this effect diminishing as the pH extends to a region where it is buffered to match the media pH (approx. >150 µm). This aspect is further emphasized in Figure S-9B, showing the fluorescence signal onset time, which is taken as the time the normalized intensity changes by 10% from that of the intensity at 0 minutes. For the targeted or neighboring cells this change occurs at the start of the delivery (2 min), with a (small) but increasing delay with further distance from the delivery point. Beyond the 130-150 µm distance, the onset time is much less consistent, with some cells surpassing

this threshold within the time of delivery (2-4 min) and some surpassing this threshold post-delivery, most probably the effect of repeated imaging.

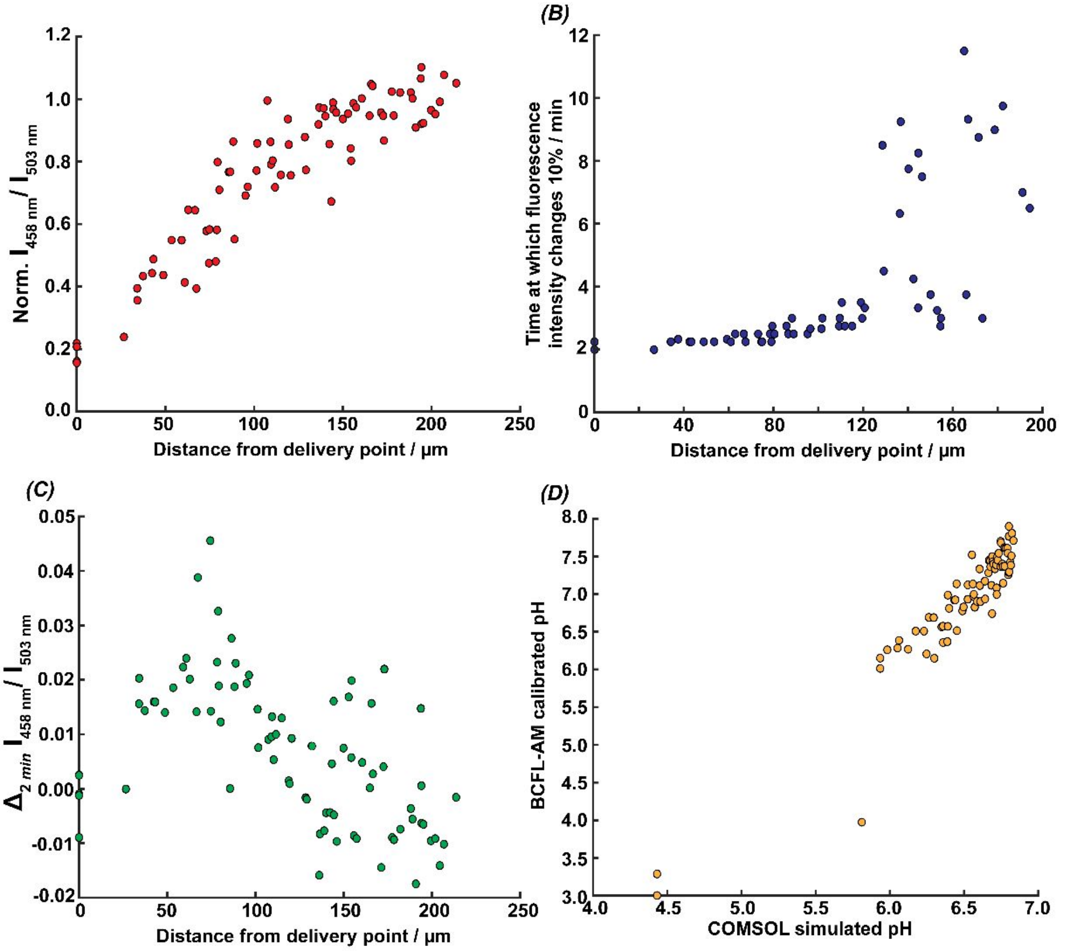

Figure S-9. *(A)* $I_{458\ nm}/I_{503\ nm}$ *as a function of the position of each cell from the delivery position studied across the experiments, as shown in Figure 5, from BCFL-AM at the end of the delivery pulse. (B) Fluorescence onset against distance from delivery point. (C)* $\Delta_{2\ min} I_{458\ nm}/I_{503\ nm}$ *post-delivery against distance from the delivery point. (D) COMSOL simulated pH, converted from each cell distance, against the experimental calibrated BCFL-AM pH, at the end of the delivery pulse.*

Figure S-9C shows the fluorescence linear rate of change across the first 2 minutes post-delivery ($\Delta_{2\ min} I_{458\ nm}/I_{503\ nm}$), demonstrating the initial rate of intracellular pH recovery after the acid challenge ceased. A positive value indicated recovery of the cell pH. While there is some variability in the cell responses, the nearest cells to the delivery site (up to 25 μm distance away) do not show pH recovery following the delivery. Most cells further from the site,

distance up to approx. 120 μm demonstrate a recovery in intracellular pH. This is reflected in Figure 4C (main text), where the neighboring cells in the targeted cell initially begin to recover pH once the stimulus is removed. Cells even further away do not show any appreciable change in signal because these are not challenged by the acid delivery. Finally, Figure S-9D shows the COMSOL simulated pH at each cell position against the BCFL-AM calibrated intracellular pH at the end of the delivery pulse, for which a correlation is evident.

SI-6 Pipette fabrication

A P-2000 Sutter Instruments laser puller was used to fabricate all probes in this work, where borosilicate glass capillaries (o.d. 1.2 mm, i.d. 0.69 mm, Harvard Apparatus) were pulled to process pipettes with a radius of approximately 1 μm. Pulling parameters:

Line 1: Heat: 350, Fil: 4, Vel: 40, Del: 200, Pull:-.

SI-7 HeLa cell culture and media preparation

HeLa cells were obtained from Public Health England, supplied by the European Collection of Authenticated Cell Cultures (ECACC, catalogue number: 93021013). Cells were grown in Minimum Essential Medium Eagle (Sigma Aldrich, M2279) supplemented with L-glutamine (used in 100-fold dilution, Sigma Aldrich, G7513), penicillin and streptomycin (used in 1000-fold dilution, Sigma Aldrich, P4333), heat-inactivated fetal calf serum (10-fold dilution, HIFC, Sigma Aldrich, 12106C), and non-essential amino acids (100-fold dilution, Sigma Aldrich, M7145). Due to the sodium bicarbonate buffer used, cells were incubated at 37˚C in 5% CO_2. Cell flasks were passaged when the confluency reached approximately 80% (no more than a week), and never exceeded a passage number of 10.

Individual sample dishes of cells were prepared on 50 mm diameter WillCo Wells (Glass thickness No. 1.5, USE, HBST-5040) the evening prior to experiments, cultures were diluted to approximately 5×10^6 cell mL^{-1}.

For SICM, where the equipment is not held in a CO_2 environment, the media was replaced with a Minimum Essential Medium (Sigma Aldrich, 56416C, referred to here at M5) buffered with HEPES (100-fold dilution, Sigma Aldrich, H0887), warmed to 37˚C.

SI-8 Confocal microscopy

Cell staining

For staining with BCFL-AM, samples were firstly gently washed with 3 × 1 mL application of sterile PBS to remove of residue media. 3 mL of warmed M5 was then added to the plate, along with 10 µL of a 3 mM BCFL-AM stock in DMSO, giving a staining concentration of 10 µM. The plate was then returned to the incubator for 30 minutes, at 37°C. The stain solution was then removed and washed with M5, and the solution replaced with fresh media for the experiment. The protocol was very similar with the pHRodo Red, but 3 µL of a 5 mM stock was taken and dissolved in 3 mL of M5 media, giving a staining concentration of 5 µM.

For WGA fluorescein, 1 mg of the WGA fluorescein was dissolved in 1 mL of PBS. The overnight sample dish was washed with 3 × 1 mL application of sterile PBS. 3 mL of warmed M5 was added to the plate, along with 60 µL of the 1 mg/mL WGA fluorescein, resulting in a staining concentration of 20 µg/mL. The plate was incubated for 10 minutes, at 37°C. After incubation, the plate was washed with M5.

Dye Calibration

For calibration of the dyes, 10 mL aliquots were prepared in sterile conditions and adjusted with HCl and NaOH stocks until their pH was within interval values between pH 4 and 9. To each, the non-cell specific stain was added, giving a final concentration of fluorescein at 2 µM or BCECF at 10 µM. The calibration was always taken on the day the aliquots were prepared.

To measure the fluorescence, each aliquot in turn was added into new WillCo wells dishes and imaged with the same conditions as used experimentally. The image plane was set to be as for the cell-inclusive experiments. Measurements were made in triplicate.

Microscope settings

Unless otherwise stated, microscope settings involved using a ×20 objective, with a zoom of 2.5, allowing a reasonably fast scanning frequency speed of 1000 Hz whilst still being able to image a wide plane to include numerous cells (512 × 512 pixels, pixel size: 606.65 × 606.65 nm). The pinhole was 84.91 µm, giving a section thickness of *ca.* 4 µm.

Cells were imaged using a time lapse study, with imaging at every 15-30 second interval (described with each figure). Short *z*-stacks of (approximately 3 frames, 2.5 μm step size) were used to capture most of the cell volume, to counter any potential *z*-drift over longer time lapses. After imaging was completed, the remaining time period was kept dark, to minimize photobleaching effects (example: intervals of 20 seconds, a 3-frame *z*-stack takes 3.2 sec, followed by 17.8 sec dark rest). Time lapses were typically begun 2 minutes before the delivery potential was applied, this allowed the collection of several images of unperturbed cells to provide an initial cell fluorescence and background. Imaging continued throughout the delivery period and then for several minutes after to capture the changing cell fluorescence.

Laser settings

For the BCFL-AM dye, the argon laser was set at 458 nm (laser power measured at *approx.* 2.05 mol photon per cm^2), and the white light laser was set to 503 nm (laser power measured at *approx.* 0.27 mol photon per cm^2). For the WGA Fluorescein dye, the argon laser was set at 488 nm (laser power measured at 10.58 mol photon per cm^2). The pHRodo Red dye required excitation at 566 nm (laser power measured at *approx.* 0.95 mol photon per cm^2).

SI-9 Image analysis

Image analysis was performed through three stages, firstly using FIJI ImageJ software with custom made scripts. Firstly, a background correction was performed to minimize any background noise in the data. Next, the *z*-stacks of the cell were grouped to present a single plane accounting for the whole cell volume, and a series of masking protocols was employed to determine which best accounted for but differentiated between cells. Each cell of the frame was then considered an individual region of interest (ROI) and numbered accordingly. The mean, min, max and standard deviation of fluorescence intensity, along with the area and centroid coordinates were then measured for each cell, across each frame of the time lapse and reported in a data file. This data file was then analyzed with MATLAB (R20201b), using a custom script which reports the data in the format seen throughout the paper.

References

(1) McPherson, I. J.; Brown, P.; Meloni, G. N.; Unwin, P. R. Visualization of Ion Fluxes in Nanopipettes: Detection and Analysis of Electro-osmosis of the Second Kind. *Anal. Chem.* **2021**, *93* (49), 16302-16307

(2) Brown, W.; Kvetny, M.; Yang, R.; Wang, G. Higher Ion Selectivity with Lower Energy Usage Promoted by Electro-osmotic Flow in the Transport through Conical Nanopores. *J. of Phys. Chem. C* **2021**, *125* (6), 3269-3276

(3) Brown, W.; Li, Y.; Yang, R.; Wang, D.; Kvetny, M.; Zheng, H.; Wang, G. Deconvolution of electroosmotic flow in hysteresis ion transport through single asymmetric nanopipettes. *Chem Sci* **2020**, *11* (23), 5950-5958

(4) Yusko, E. C.; An, R.; Mayer, M. Electroosmotic Flow Can Generate Ion Current Rectification in Nano- and Micropores. *ACS Nano* **2010**, *4* (1), 477-487

(5) Goldberg, R. N.; Kishore, N.; Lennen, R. M. Thermodynamic Quantities for the Ionisation Reactions of Buffers. *J. Phys. Chem. Ref. Data* **2002**, *31*, 231-370

(6) Teahan, J.; Perry, D.; Chen, B.; McPherson, I. J.; Meloni, G. N.; Unwin, P. R. Scanning Ion Conductance Microscopy: Surface Charge Effects on Electroosmotic Flow Delivery from a Nanopipette. *Anal. Chem.* **2021**, *93* (36), 12281-12288
